# Synergistic Ion Transport and Spatial Confinement in Sb‐Embedded Hollow Carbon Nanofibers for Stable Na Metal Anodes

**DOI:** 10.1002/advs.202521115

**Published:** 2026-04-07

**Authors:** Feng Han, Menghuan Yuan, Hui Wang, Baitao Liu, Dezhi Kong, Yongtao Tian, Xinjian Li, Ye Wang, Tingting Xu, Hui Ying Yang

**Affiliations:** ^1^ Key Laboratory of Material Physics Ministry of Education School of Physic Zhengzhou University Zhengzhou China; ^2^ College of Design and Engineering National University of Singapore Singapore Singapore

**Keywords:** in situ characterizations, Na metal anode, Sb@HCF, sodiophilic, theoretical simulation

## Abstract

Sodium metal anodes (SMAs) are pivotal for developing high‐energy‐density sodium metal batteries (SMBs) but are plagued by uncontrollable dendrite growth and unstable solid‐electrolyte interphases (SEI). While 3D core‐shell hosts can mitigate these issues, these conventional designs often lack active control over ion transport and involve complex syntheses. Herein, we develop an sodiophilic 3D Sb‐embedded hollow carbon nanofiber (Sb@HCF) host via electrospinning and wet etching approach. Combining in situ characterizations and theoretical simulations, it was clearly verified that the in situ formed Na‐Sb alloy not only guides uniform Na deposition but also serves as Na^+^ transport highways, thereby significantly suppressing Na dendrite and promoting the formation of a robust NaF‐rich SEI. As a result, the Na||Sb@HCF delivers an exceptional Coulombic efficiency of 99.88% over 1400 cycles at 4 mA cm^−2^/4 mAh cm^−2^, and the symmetric cells simultaneously operate steadily over 1200 h at 10 mA cm^−2^/2 mAh cm^−2^, achieving a superior cumulative plating capacity of 6 Ah cm^−2^. Moreover, the Na@HCF||Na_3_V_2_(PO_4_)_3_@C full cell retains a capacity of 84.10 mAh g^−1^ with a Coulombic efficiency of 98.05% even after 1000 cycles. Our strategy paves a promising way for for stabilizing SMA and developing high‐energy‐density SMBs.

## Graphical abstract

This work presents a 3D Sb‐embedded hollow carbon nanofiber (Sb@HCF) host for ultra‐stable Na metal anodes. Experimental analyses and theoretical simulations clearly uncovers that in situ formed Na‐Sb alloy guides uniform Na deposition and serves as efficient ion transport highways. The Sb@HCF‐based cells achieve exceptional cycling stability in half‐, symmetric and full‐cell configurations, demonstrating a promising strategy for high‐energy‐density Na batteries.

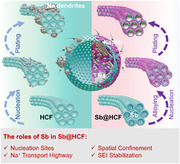

## 1 Introduction

The rapidly growing demand for portable electronics and grid‐scale energy storage has driven the need for rechargeable batteries that offer higher energy density and lower cost than current lithium‐ion technologies. Among them, Sodium (Na) metal batteries (SMBs) are promising candidates owing to the exceptional natural abundance, high theoretical capacity (1166 mAh g^−1^) and a low redox potential (−2.714 V vs SHE) of Na metal anode (SMA) [1, 2, 3, 4, 5]. However, its practical implementation faces significant hurdles, which stemming from the following inherent challenges: uncontrolled growth of Na dendrites, continuous solid‐electrolyte interphase (SEI) breakdown and reformation, and deleterious side reactions with electrolytes. These issues collectively lead to rapid capacity fade, diminished Coulombic efficiency (CE), cell's failure and ultimate safety concerns [6, 7, 8].

To overcome these limitations, diverse strategies have been adopted, such as fabricating 3D Na host [9, 10], engineering artificial SEI [11, 12], designing advanced electrolyte with functional additives [13], and exploitating advanced solid state electrolytes [14]. Notably, the 3D core‐amorphous carbon shell design featuring rich sodiophilic nucleation sites has shown promise for constructing advanced Na metal hosts. In such architectures, the sodiophilic core promotes spatially controlled and homogeneous Na plating, while the amorphous carbon confinement layer suppresses side reactions and enhances cycling reversibility. Consequently, hollow amorphous carbon shells incorporating sodiophilic agents (e.g., Sn [15], Zn [16], Bi [17], Sb [18, 19], Ga [20], Au [21], nanoparticles or oxygen/nitrogen/sulfur‐containing functional groups) [22, 23, 24] have demonstrated high CE and enhanced cycling stability. Importantly, metal (e.g., Zn [25], Sn [26], Bi [27], and Sb [28]) can chemically react with Na to form Na‐metal alloys within the core. These alloys not only guide uniform Na deposition but can also serve as efficient Na^+^ diffusion highways. Nevertheless, within conventional core‐shell hosts, these sodiophilic cores typically function only as static nucleation sites, failing to actively accelerate Na^+^ flux uniformity. Furthermore, novel core‐shell configurations, particularly those requiring carbon encapsulation of the sodiophilic core, often involves complex synthesis steps. These limitations highlight the need for simple strategies to design core‐shell architectures that fully leverage the ion‐transport superiority of Na‐metal alloys.

Sb presents a compelling solution due to its exceptional sodiophilicity and ability to form Na_3_Sb alloys, which are dual‐function materials proven to guide Na deposition and enable rapid Na^+^ transport [29, 30]. While Sb@carbon spheres have been explored, their powder‐based forms suffer from detachment from current collectors and underutilized ion‐transport capabilities. This underscores the necessity for 3D core‐shell hosts integrating structural integrity with efficient charge transport [31, 32, 33]. Herein, we fabricate a 3D hollow amorphous carbon nanofiber embedded with Sb nanoparticles (Sb@HCF) via electrospinning and wet etching. The uniformly dispersed Sb nanoparticles within the cavities and carbon matrix undergo in situ transformation into Na‐Sb alloy during Na plating, enabling dual functional roles. Specifically, the cavity‐confined Na‐Sb alloy spatially regulate Na plating, while the carbon‐embedded Na‐Sb alloy create efficient ion‐diffusion highways. This unique architecture simultaneously mitigates stress from volume expansion and stabilizes the SEI film. These synergistic mechanisms contribute to exceptional cycling stability across multiple cell configurations. As anticipated, the as‐prepared Sb@HCF host demonstrates a stable lifespan of 1400 cycles at 4 mA cm^−2^/4 mAh cm^−2^ in half‐cells, 1200 h at 10 mA cm^−2^/2 mAh cm^−2^ in symmetric cells, and over 800 cycles in full‐cell configuration paired with Na_3_V_2_(PO_4_)_3_@C (NVP@C) cathode. Overall, the low‐cost, scalable Sb@HCF design provides a suitable strategy for advanced SMBs and other battery systems.

## 2 Results and Discussion

### 2.1 Fabrication and Characterization of Sb@HCF

**FIGURE 1:**
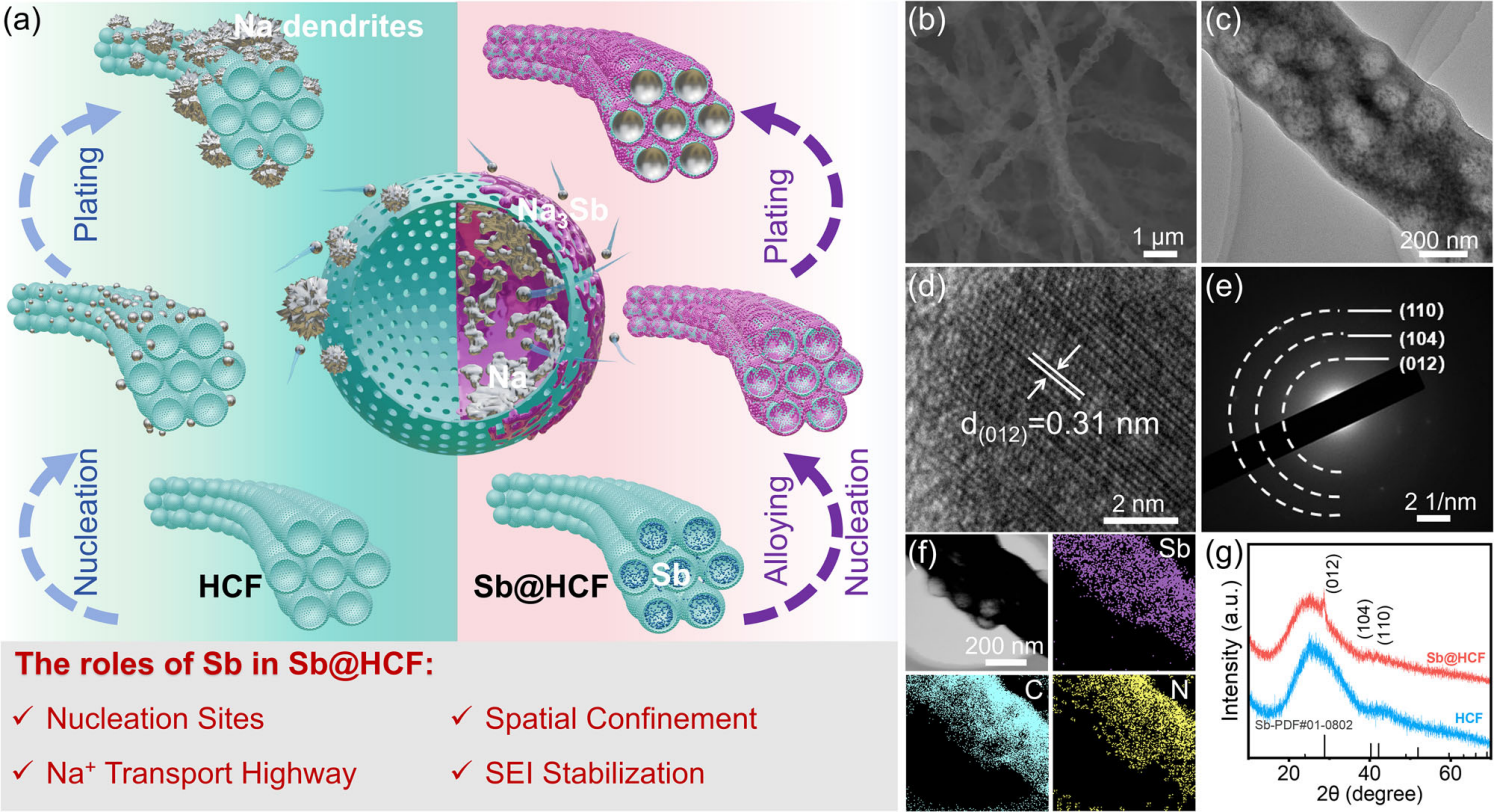
Structural and compositional characterization. (a) Schematic Na deposition behavior comparison between Sb@HCF and HCF. (b) SEM images showing the 3D fibrous architecture of Sb@HCF. (c) TEM revealing hollow cavities and uniform distribution of Sb nanoparticles. (d) HRTEM of the Sb crystallites. (e) SAED pattern of the Sb@HCF. (f) EDS elemental mapping images of Sb, C and N in Sb@HCF. (g) XRD patterns of Sb@HCF and HCF.

The Sb@HCF fabrication process (Figure S1) involves sequential electrospinning, stabilization, carbonization, and wet‐etching. Notably, the spun polymer exhibits paper‐like flexibility, as demonstrated by folding into a heart shape (Figure S2). This superior flexibility enables robust mechanical stability in the final Sb@HCF architecture. As illustrated in Figure 1a, the embedded Sb nanoparticles, which is uniformly dispersed within hollow cavities of the carbon matrix, undergo in situ transformation into Na‐Sb alloys during Na plating. Consequently, the cavity‐confined alloys act as nucleation sites to preferentially guide Na deposition into the hollow cavities, while carbon‐embedded alloys create efficient highways for Na^+^ diffusion [34]. This spatial control of Na deposition isolates the deposited Na metal from electrolyte via the alloy‐integrated carbon wall, restricting SEI formation to the outer surface. Simultaneously, the hollow structure accommodates volume changes during cycling, maintaining structural integrity, while the stable SEI layer prevents dendrite formation and interface degradation [35]. The interconnected mechanisms of spatially confined deposition, enhanced ion transport, volume buffering and interfacial stabilization enable exceptional electrochemical performance. In sharp contrast, the bare HCF suffers from random Na deposition, uncontrolled dendrite growth and severe SEI fracturing, highlighting the critical role of Sb in stabilizing SMA.

Scanning Electron Microscopy (SEM) characterization confirms that the synthesized Sb@HCF nanofibers contains interconnected hollow spherical cavities (Figure 1b), which demonstrates excellent electrolyte wettability as evidenced by contact angle test (Figure S3), hence effectively accelerating Na^+^ transport. The transmission electron microscopy (TEM) analysis reveals Sb nanoparticles uniformly dispersed within cavities and the amorphous carbon matrix (Figure 1c). High resolution TEM imaging (Figure 1d) reveals distinct lattice fringes with an interplanar spacing of 0.31 nm, which can be indexed to the (012) plane of rhombohedral Sb (JCPDS 01–0802) [36]. This finding is further corroborated by the selected area electron pattern (SAED) in Figure 1e. Additionally, the energy dispersive X‐ray spectroscopy (EDS) elemental mapping of Sb@HCF demonstrates homogeneous distribution of C, N, and Sb (Figure 1f). The N in Sb@HCF originates from the carbonization of the precursor polyacrylonitrile (PAN). The EDS analysis confirms the absence of Si residue and the complete removal of the SiO_2_ template in the final Sb@HCF. The very low Si signal in the etched Sb@HCF (0.43 at%) is comparable to the background level of the blank carbon film (0.27 at%), which is attributable to the intrinsic detector background of the EDS system(Figure S4). The Sb content in Sb@HCF was determined to be 7.55 wt.% through thermogravimetric analysis (TGA) (Figure S5). X‐ray diffraction (XRD) (Figure 1g) shows peaks at 28.7°, 40.1°, and 42.0° assigned to (012), (104), and (110) planes of rhombohedral Sb, confirming complete reduction of SbCl_3_ [37]. Broad peaks at ∼26° and 43° originate from amorphous carbon in both Sb@HCF and HCF. The Raman spectra indicate that Sb@HCF possesses a higher I_D_/I_G_ ratio than HCF (1.16 vs 1.03), suggesting increased carbon disorder due to Sb incorporation, which will enhance active sites for Na plating (Figure S6). The N_2_ adsorption‐desorption shows Sb@HCF has a lower specific surface area (166.83 m^2^ g^−1^) than HCF (660.65 m^2^ g^−1^), attributable to Sb nanoparticle occupancy (Figure S7). X‐ray photoelectron spectroscopy (XPS) was employed to analyze the surface chemistry of Sb@HCF and HCF (Figures S8 and S9) [38]. The deconvolution of the C 1s XPS spectra for both Sb@HCF and HCF reveals three distinct components, assigned to C═O, C─O and C─C/C═C functional groups [39]. The N 1s spectra display characteristic binding energies indicative of graphitic N (402.3 eV), pyrrolic N (400.7 eV) and pyridinic N (398.2 eV).

### 2.2 Electrochemical Performance Evaluation

**FIGURE 2:**
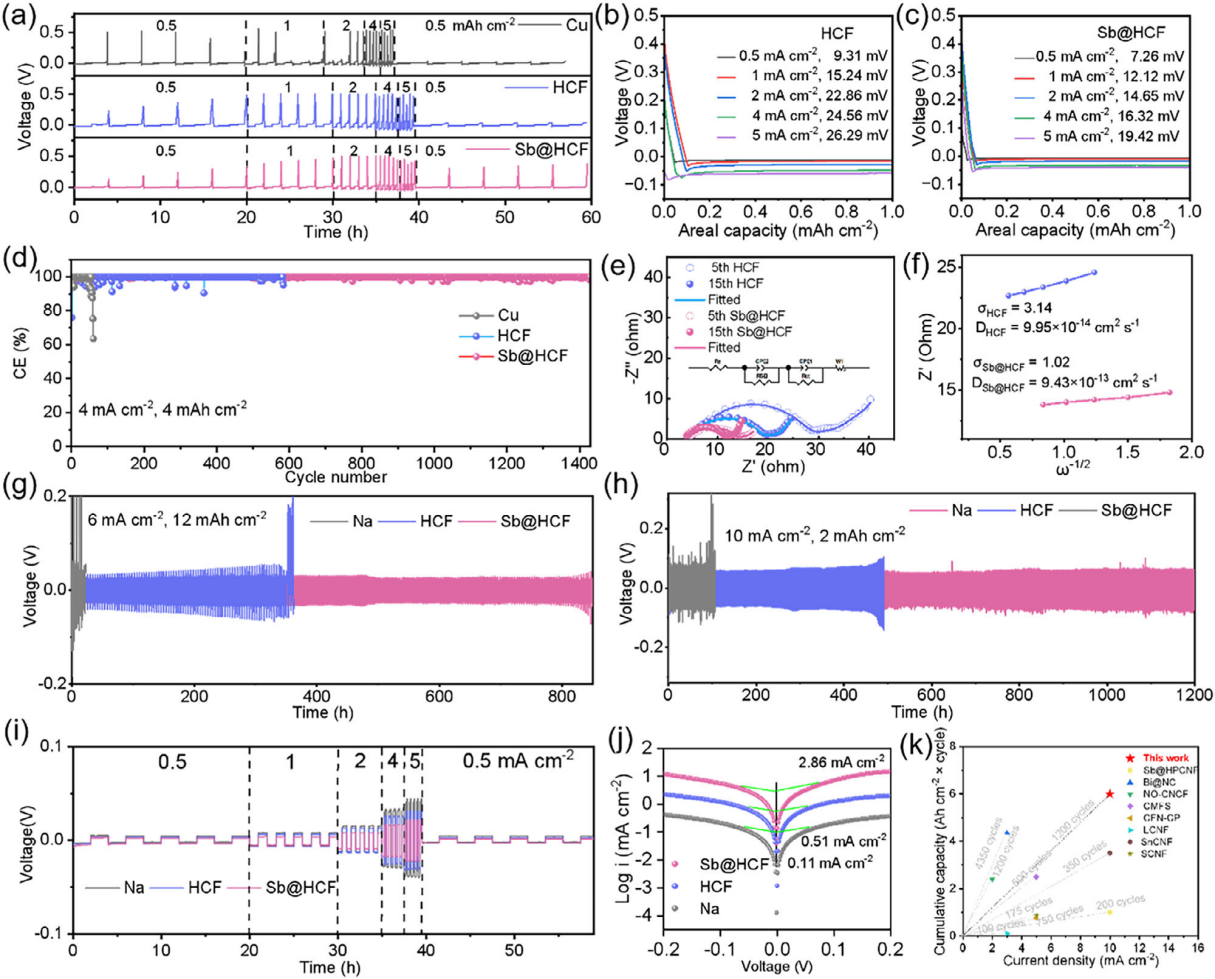
Electrochemical performance evaluation of Cu, HCF, and Sb@HCF electrodes. (a) Rate performances of Cu, HCF, and Sb@HCF electrodes at 0.5, 1, 2, 4, 5 mA cm^−2^. Voltage profiles of (b) HCF and (c) Sb@HCF electrodes at various current densities. (d) CEs of the Cu, HCF, and Sb@HCF electrodes at 4 mA cm^−2^ with a capacity of 4 mAh cm^−2^. (e) Nyquist plots and fitted curves (solid line) of Sb@HCF (red) and HCF (blue) electrodes after 5th (hollow dots) and 15th cycles (solid dots). (f) Diffusion coefficients of Sb@HCF and HCF electrodes calculated after 15 cycles. Galvanostatic discharge/charge potential profiles in symmetric cells at (g) 6 mA cm^−2^ with a capacity of 12 mAh cm^−2^, (h) 10 mA cm^−2^ with a capacity of 2 mAh cm^−2^. (i) Rate performance of symmetric cells at various current densities. (j) Tafel curves in symmetric cells. (k) Comparison between the cumulative capacities of symmetric cells at different current densities based on various core‐shell host incorporating sodiophilic sites.

The rate capabilities of Cu, HCF, and Sb@HCF electrodes were evaluated in asymmetric cells (Figure 2a). At a current density of 0.5 mA cm^−2^, the Cu electrode demostrates a notably high nucleation overpotential of 28.84 mV and further suffers short‐circuiting at 1 mA cm^−2^ (Figure S10). The HCF electrode, on the other hand, shows a nucleation overpotential of 9.31 mV at 0.5 mA cm^−2^, 15.24 mV at 1 mA cm^−2^, 22.86 mV at 2 mA cm^−2^, 24.56 mV at 4 mA cm^−2^, and 26.69 mV at 5 mA cm^−2^, respectively (Figure 2b). The Sb@HCF electrode exhibits significantly lower and more stable overpotential of 7.26 mV at 0.5 mA cm^−2^, 12.12 mV at 1 mA cm^−2^, 14.65 mV at 2 mA cm^−2^, 16.32 mV at 4 mA cm^−2^, respectively (Figure 2c). These minimal overpotentials confirm Sb@HCF's exceptional rate performance (Figure S11), demonstrating that the existence of Sb nanoparticles significantly reduces the nucleation barrier during Na deposition [40]. Furthermore, Na||Sb@HCF half‐cells maintain a high average CE as high as 99.88% across 1400 cycles under a current density of 4 mA cm^−2^ and an areal capacity of 4 mAh cm^−2^, delivering outstanding cycling stability (Figure 2d; Figure S12). In stark contrast, Na||HCF cells show an inferior average CE of 94.05% and voltage fluctuations after 600 cycles, and Na||Cu cells fail within 40 cycles due to severe polarization.

To gain deeper insights into interfacial stability and charge transfer kinetics, charge transfer resistance (*R*_ct_) was evaluated using electrochemical impedance spectroscopy (EIS). Nyquist plots for the Sb@HCF and HCF electrodes were fitted before cycling and after 5 and 15 cycles at a current density of 1 mA cm^−2^ and a capacity of 1 mAh cm^−2^ (Figure 2e), and the equivalent circuit used to fit the data was shown in the inset. Before cycling, the *R*_ct_ values are 319.8 Ω for Sb@HCF, 639.5 Ω for HCF (Figure S13 and Table S1). After 5 and 15 cycles, the Sb@HCF electrode exhibits significantly lower *R*_ct_ values (3.2 and 1.9 Ω, respectively) compared to the HCF electrode (4.1 and 2.4 Ω) (Table S2). The lower *R*_ct_ of Sb@HCF indicates faster charge transfer kinetics, which is further supported by the calculated higher Na^+^ diffusion coefficients (*D*) for Sb@HCF (9.43 × 10^−13^ cm^2^ s^−1^) than HCF (9.95 × 10^−14^ cm^2^ s^−1^) (Figure 2f). The diffusion coefficient was calculated according to following equation [41]:

(1) $$D=\frac{R^{2}T^{2}}{2A^{2}n^{4}F^{4}C^{2}\sigma^{2}}$$

where R denotes the gas constant (8.31 J mol^−1^ K^−1^), T represents the absolute temperature set at 298 K, A corresponds to the electrode surface area, n indicates the number of electrons transferred per redox event, F stands for the Faraday constant (9.65 × 10^4^ C mol^−1^), C signifies the concentration of Na^+^ ions, and σ is the Warburg factor.

Symmetric cells (Na||Na, HCF||HCF, Sb@HCF||Sb@HCF) were assembled to evaluate electrochemical performance. At 6 mAh cm^−2^ with a high areal capacity of 12 mAh cm^−2^, the Sb@HCF||Sb@HCF cell maintains a low voltage hysteresis of 30 mV over 900 h (Figure 2g). In contrast, The HCF||HCF cell only cycles for 370 h, and exhibits increasing hysteresis, which increased from an initial 33 to 54 mV at 370 h and accompanied by severe fluctuations before failure. The Na||Na cell fails after only 23 h with a much higher hysteresis of about 60 mV, attributed to SEI destruction and dead Na formation. Notably, when tested under harsher conditions (10 mA cm^−2^, 2 mAh cm^−2^), Sb@HCF||Sb@HCF demonstrates exceptional stability for 1200 h with a low voltage hysteresis of 58 mV. Meanwhile, HCF||HCF and Na||Na cells exhibit rising hysteresis and ultimately fail after 470 and 100 h (Figure 2h). Rate performance further verifies the performance superiority of the Sb@HCF||Sb@HCF over HCF||HCF and Na||Na (Figure 2i). Tafel plot analysis reveals that the Sb@HCF electrode exhibits a superior exchange current density of 2.86 mA cm^−2^, significantly exceeding that of HCF (0.51 mA cm^−2^) and Na (0.11 mA cm^−2^) (Figure 2j; Figure S14). The performance enhancement of Sb@HCF||Sb@HCF cell verifies the function of Sb in effectively suppressing dendrite growth and enhancing interfacial ion transport kinetics. Furthermore, the Sb@HCF electrode demonstrates superior cumulative capacity of 6 Ah cm^−2^ at high current density among core‐shell host structures incorporating sodiophilic sites (Figure 2k and Table S3).

### 2.3 Na Metal Dynamic Deposition Behavior

**FIGURE 3:**
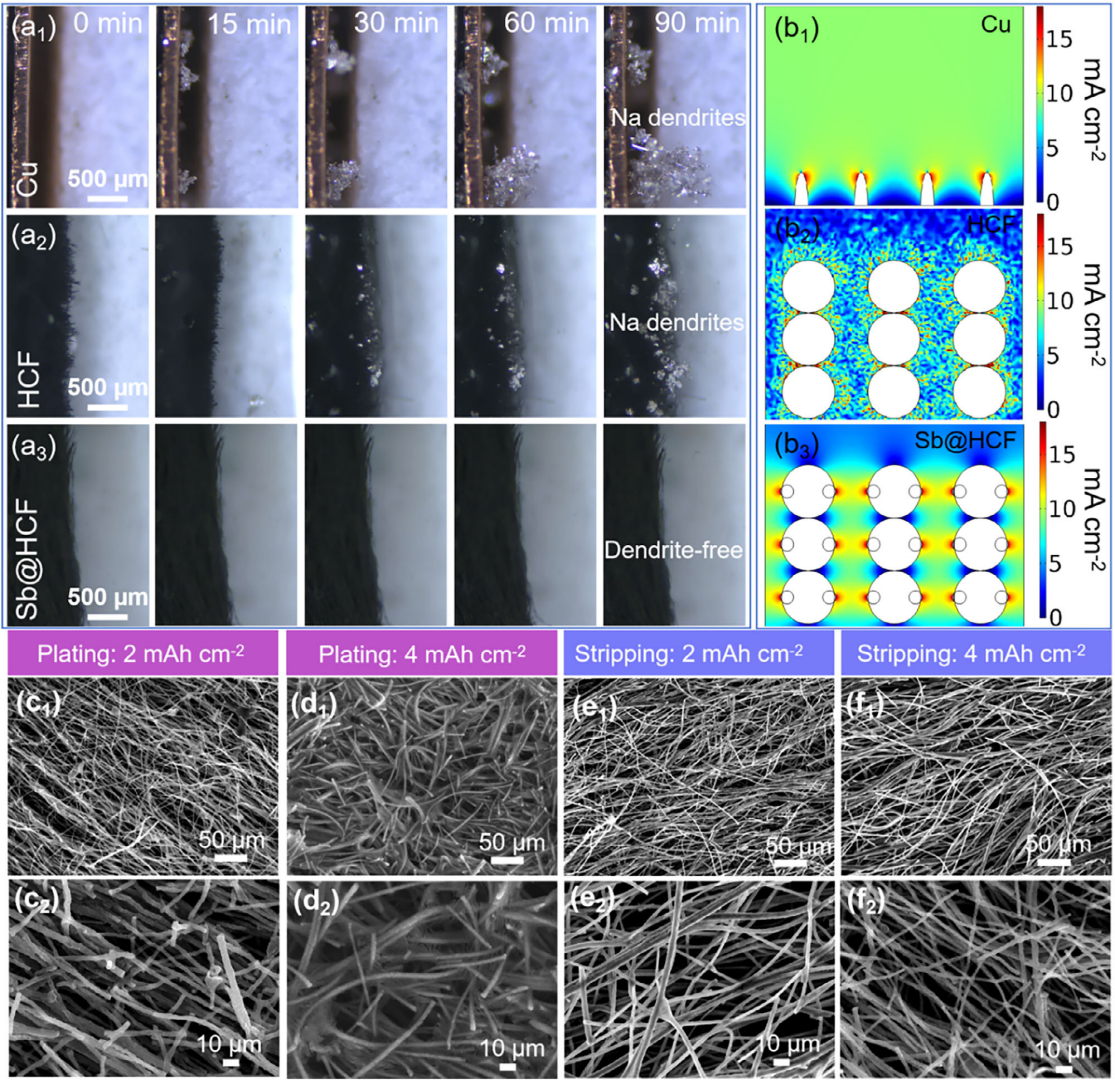
Na deposition behavior and morphological evolution in various electrodes of Cu, HCF, and Sb@HCF. (a) In situ optical photographs of Na deposition on Cu, HCF, and Sb@HCF at current density of 2 mA cm^−2^. (b) COMSOL simulations of surface current density distribution on Cu, HCF, and Sb@HCF electrodes. Ex situ SEM characterization of Sb@HCF at a plating capacity of (c_1_,c_2_) 2 mAh cm^−2^, (d_1_,d_2_) 4 mAh cm^−2^, and a stripping capacity of (e_1_,e_2_) 2 mAh cm^−2^, (f_1_,f_2_) 4 mAh cm^−2^.

In situ optical microscopy was employed to monitor the dynamic deposition behavior of Na metal under 2 mA cm^−2^. The Na dendrites gradually occur upon Cu electrode within 15 min, and then Na dendrites cover the electrode surface, ultimately causing short‐circuit by 90 min (Figure 3a). The HCF electrode shows dendritic growth after 30 min with significant Na dendrite accumulation at 90 min. Conversely, the Sb@HCF electrode surface always maintains uniform, dendrite‐free plating throughout the 90‐min observation. Furthermore, COMSOL simulations corroborate these findings (Figure 3b). The Cu electrode shows protruding tips with concentrated current density, while HCF demonstrates moderate nucleation improvement. Critically, Sb@HCF achieves homogeneous deposition due to abundant nucleation sites around Sb nanoparticles. The morphological evolution of Sb@HCF, HCF and Cu electrode during Na deposition/stripping was further observed by using ex situ SEM (Figure 3c–f). When the Na metal is plated to 2 mAh cm^−2^, the Sb@HCF fiber encapsulates Na metal within core‐shell structures (Figure 3c), while HCF forms external Na layers and Cu displays uneven surface deposition (Figures S15a and S16a). At a plating capacity of 4 mAh cm^−2^, Sb@HCF still maintains uniform Na distribution (Figure 3d), whereas HCF shows irregular deposits and Cu exhibits dendrites with dead Na (Figures S15b and S16b). Following stripping from 2 to 4 mAh cm^−2^, the Sb@HCF electrode surface reemerges smoothly without Na residue (Figure 3e), while HCF fibers retains uneven Na metal deposits (Figure S15c,d) and Cu substrate exhibits large residual Na metal chunks (Figure S16c,d).

### 2.4 Na Metal Plating/Stripping Mechanism

**FIGURE 4:**
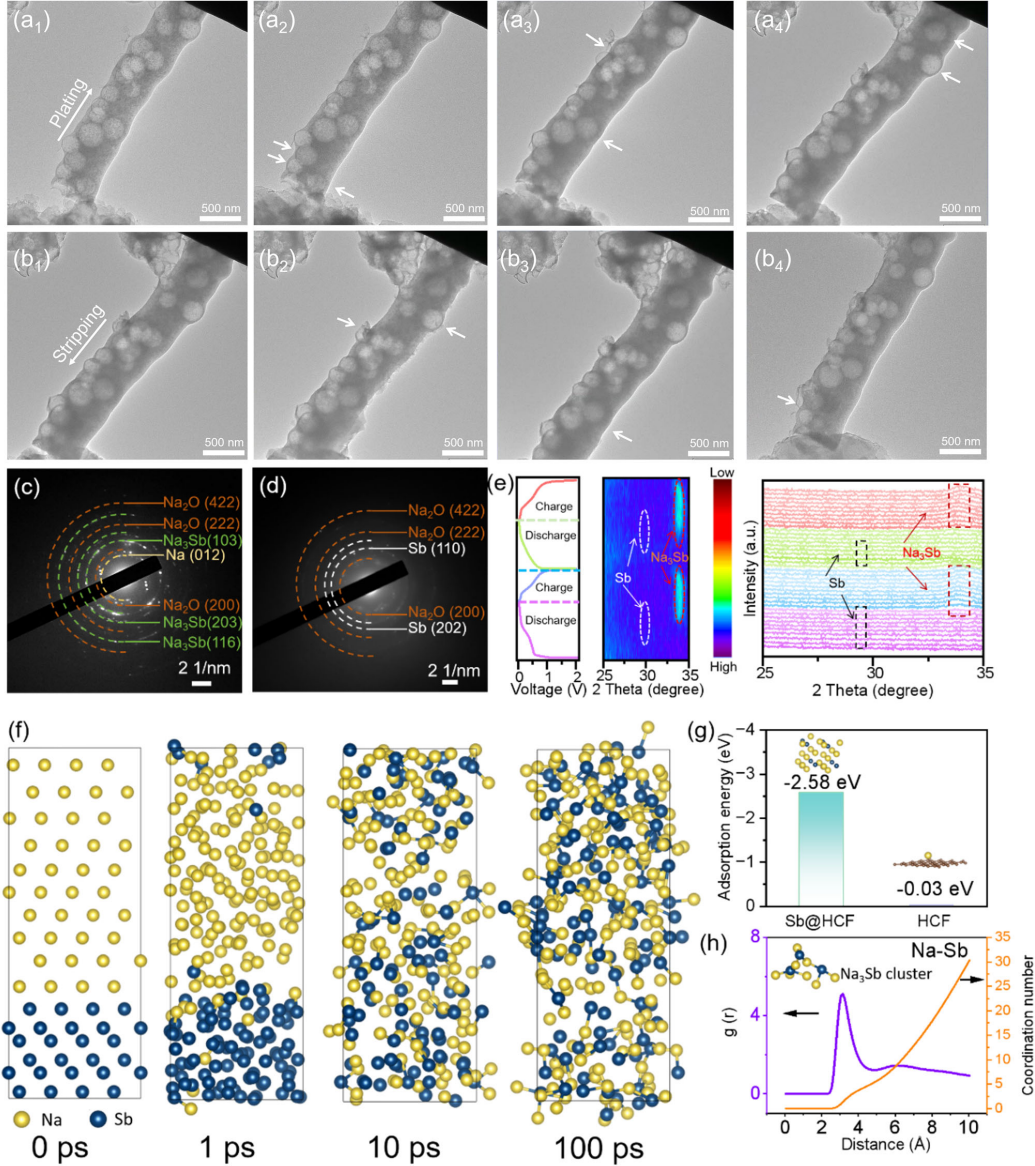
In situ TEM and XRD analysis of Na plating/stripping mechanisms in Sb@HCF. In situ TEM snapshots capturing the dynamic (a) Na plating and (b) stripping process within the Sb@HCF nanofiber, with arrows indicating the plating/stripping front. The SAED patterns (c) after plating and (d) post‐stripping. (e) Galvanostatic voltage profile and corresponding in situ XRD contour map. (f) AIMD simulation snapshots. (g) The adsorption energies of Na^+^ on Sb@HCF and HCF.(h) the RDFs and the corresponding coordination number.

To elaborate the Na metal plating/stripping mechanisms within Sb@HCF, the real‐time Na metal plating and stripping processes in the Sb@HCF were detected by in situ TEM [42, 43]. The Sb@HCF working electrode, Na metal counter electrode and Na_2_O solid electrolyte were assembled as experimental setup (Figure S17). Under a 3 V bias with a physical contact between Sb@HCF and Na_2_O, Na deposition initiates immediately, triggering simultaneous Sb volume expansion and alloying (Figure 4a). Subsequently, Na metal progressively fully fills the Sb@HCF nanofiber within 6 min, under the guidance of Na‐Sb alloy (Figure 4a). During stripping under −3 V bias, Na removal progresses, culminating in full extraction at 12 min (Figure 4b). Phase evolution was also corroborated by SAED patterns of Sb@HCF in its pristine [44], post‐plating and post‐stripping states (Figure 4c,d; Figure S18). Pristine Sb@HCF (Figure S18) transforms to coexisting Na/Na_3_Sb after plating (Figure 4c), while post‐stripping analysis confirms the disappearance of Na and Na_3_Sb and the regeneration of Sb (Figure 4d). Complementary in situ XRD tracked dynamic phase evolution during cycling (Figure 4e). Initial discharge is triggered by progressive attenuation of Sb (012) peak at 28.7° and concurrent emergence of the Na_3_Sb (110) peak at 33.4°. Complete transformation to Na_3_Sb occurrs at deep discharge, accelerating Na^+^ transport. Subsequent charging reverses this process, and the diminished Na_3_Sb (110) peaks signify the dealloying stage, followed by the reformation of Sb (012) peak [45]. The reaction can be described by the following equation [46]:

(2) $$\begin{array}{ccc} \mathrm{Sb}+(3-x)Na^{+}+(3-x)e^{-} & & \Leftrightarrow Na_{\left(3-x\right)}\mathrm{Sb}+\mathrm{xN}a^{+}+xe^{-}\\ & & \Leftrightarrow Na_{3}\mathrm{Sb}\;(0<x<3) \end{array}$$

Importantly, the dealloying of Na_3_Sb back to Sb requires a voltage above 1 V, whereas the electrochemical testing of the Na metal anode operates below 0.5 V. Thus, the Na_3_Sb alloy remains electrochemically stable within the operating voltage window of the Na metal anode and continuously functions as a permanent sodiophilic nucleation site. Ab initio molecular dynamics (AIMD) simulations were performed to investigate Na affinity and diffusion kinetics [47]. The simulations show that a Na‐Sb alloy cluster forms rapidly within 10 ps, with more Na atoms continuously aggregating between 10 and 100 ps (Figure 4f). The trajectories of Na^+^ also confirm the superior Na^+^ transport on the Na_3_Sb alloy compared to the HCF matrix (Figure S19). This rapid aggregation demonstrates both strong sodiophilicity and fast Na diffusion kinetics within the alloy. The strong affinity is quantitatively supported by binding energy calculations, which yield a value of −2.58 eV for Na_3_Sb vs only −0.03 eV for the HCF matrix (Figure 4g). Based on the simulated trajectory, we further calculated the radial distribution function (RDF) and the corresponding coordination number (Figure 4h). The RDF curve exhibits a prominent main peak at 3.15 Å, indicating that Na and Sb atoms are closely packed and stably coordinated in the Na‐Sb alloy, which aligns well with in situ XRD results. This enhanced diffusion kinetics is critical for the high‐rate performance of the Sb@HCF electrode, as the dynamically formed Na‐Sb alloy facilitates rapid Na^+^ transport even under high current densities.

### 2.5 Composition and Structure of SEI

**FIGURE 5:**
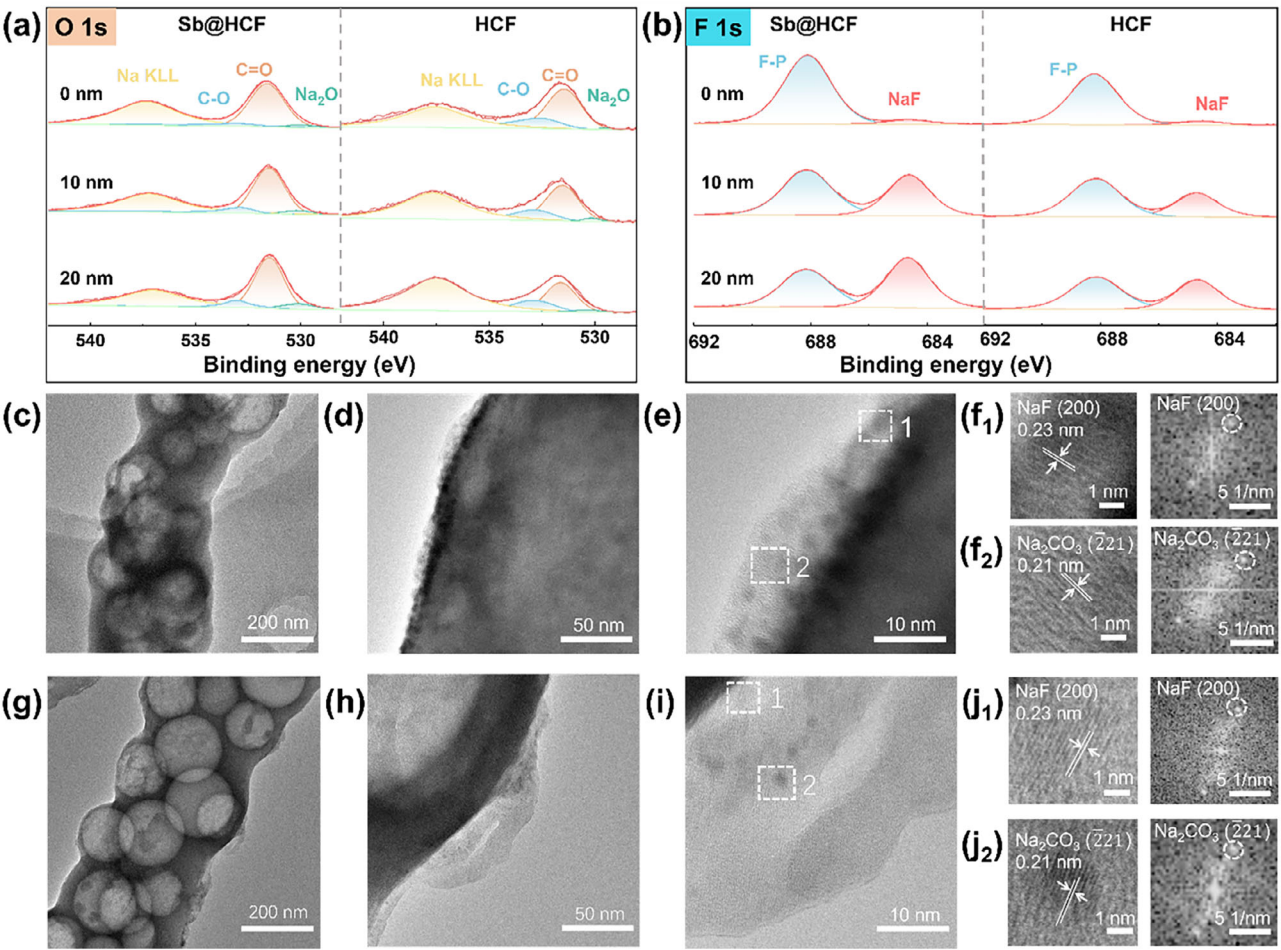
Comparative analysis of SEI composition and morphology in Sb@HCF vs HCF electrodes after 15 cycles under 1 mA cm^−2^/1 mAh cm^−2^. In‐depth XPS spectra of (a) O 1s and (b) F 1s. The Cryo‐TEM image (c–e), HRTEM and FFT (f) of SEI layer in Sb@HCF after 15 cycles. The Cryo‐TEM image (g–i), HRTEM and FFT (j) of SEI layer in HCF after 15 cycles.

To further elucidate the role of Sb alloy in stabilizing the Na metal interface, Depth‐profiling XPS analysis was performed on Sb@HCF and HCF electrodes to characterize the evolution of SEI's chemistry composition. In the near‐surface region, the O 1s spectra for both electrodes reveals two characteristic peaks at 532.9 eV (C─O) and 531.5 eV (C═O) for organic species (Figure 5a), originating from electrolyte reduction [48]. The intensity of these organic components diminishes with depth for both samples, and the Sb@HCF electrode exhibits a significantly lower organic content at equivalent depths compared to the HCF control, a finding corroborated by C 1s analysis (Figure S20). Concurrently, the inorganic SEI component strengthens markedly in the Sb@HCF electrode. The O 1s spectra confirm a pronounced increase in the Na_2_O signal with depth. Furthermore, the F 1s spectra demonstrate a substantial enrichment of NaF (684.6 eV) within the crucial 0–20 nm depth region. A direct comparison shows the NaF peak intensity is significantly stronger in Sb@HCF than in HCF (Figure 5b). In the quantitative analysis, the Sb@HCF electrode exhibits a consistently lower content of organic species (C─O/C═O) and a progressively higher content of inorganic constituents (NaF) compared to the HCF control(Figure S21). Collectively, these results indicate the formation of a more inorganic‐rich SEI structure with beneficial Na_2_O and NaF in Sb@HCF electrodes, and the SEI structure is characterized by a clear transition from an organic‐dominated outer layer to an inorganic‐rich inner layer. Furthermore, Cryo‐TEM investigations demonstrate a uniform and compact SEI film in Sb@HCF electrode (Figure 5c–f). The HRTEM and the FFT identify crystalline domains with lattice spacings of 0.21 and 0.23 nm, attributed to Monoclinic Na_2_CO_3_ ($\bar{2}$21) (JCPDS No. 00‐019‐1130) and NaF (200) planes (JCPDS No. 96‐101‐1142), respectively [49] (Figure 5f). This dense inorganic‐rich SEI structure functions as an effective physical barrier, enabling to prevent parasitic reactions and maintain structure integrity of SEI during cycling. In stark contrast, HCF electrodes develop thicker and heterogeneous SEI structures (Figure 5g–j), accelerating SEI degradation and inducing Na dendrite growth. After 75 cycles, the SEI on the Sb@HCF electrode remains uniform and thin, in contrast to the noticeably thicker and more irregular SEI formed on the HCF electrode (Figure S22). The formation of a spatially heterogeneous SEI in HCF can be attributed to the less‐guided Na^+^ flux and the localized, uneven electrolyte decomposition.

### 2.6 Na Metal Full Cell and Pouch Cell

**FIGURE 6:**
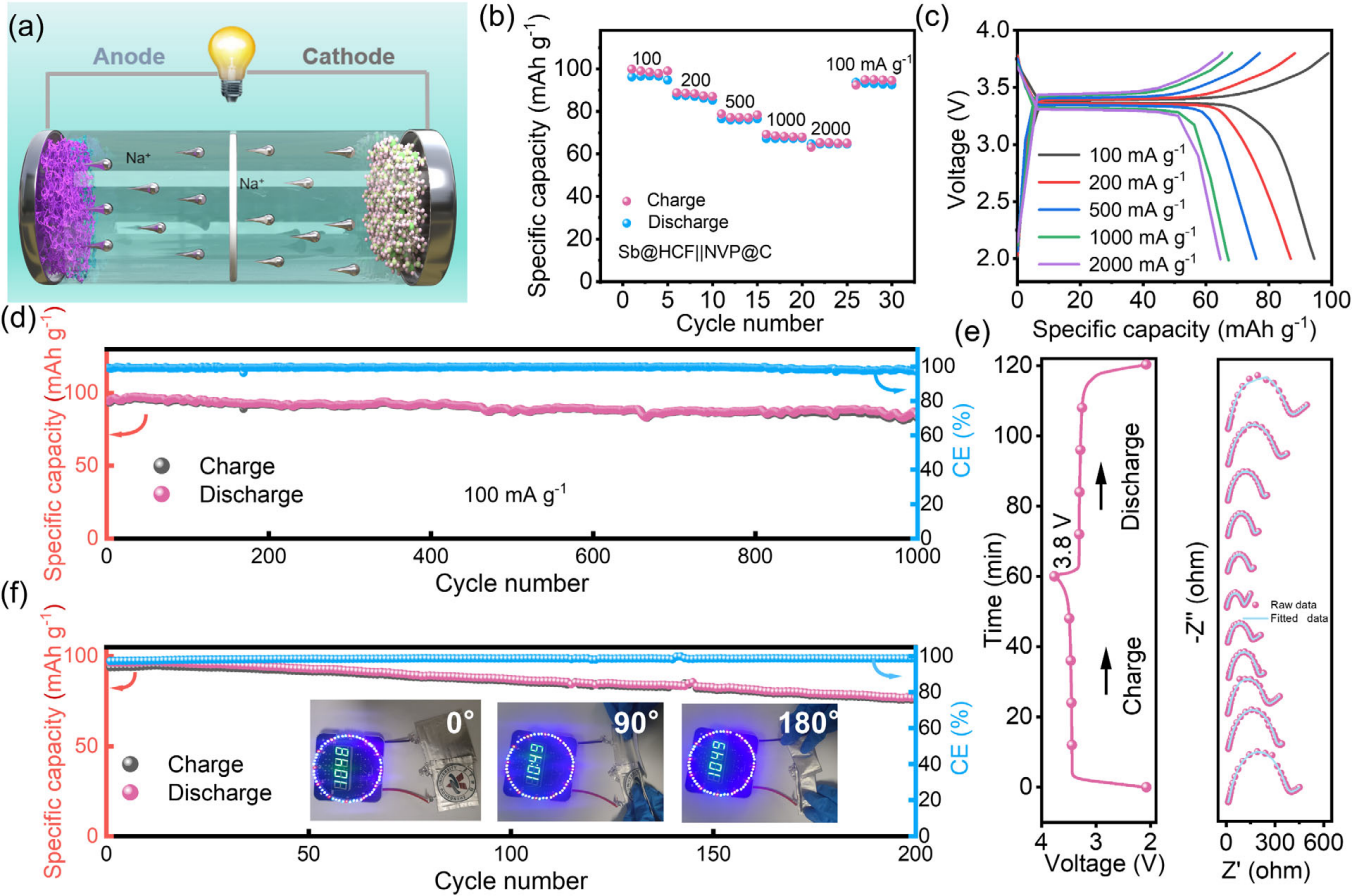
Full‐cell performance and practical demonstration. (a) Schematic illustration of the Na@Sb@HCF||NVP@C full cell. (b) Rate performance and (c) the corresponding GCD curves at the current densities from 100 to 2000 mA g^−1^. (d) Cycling performance with related CE at 100 mA g^−1^. (e) In situ EIS evolution of the Na@Sb@HCF||NVP@C full cells, with the experimental data and fitted curves shown as scatter points and solid lines, respectively. (f) Long‐term cycling stability and corresponding CE of flexible Na@Sb@HCF||NVP@C pouch cells, and the powering of an electronic clock under various folded states.

The Na@Sb@HCF anode and NVP@C cathode were assembled into a full cell to investigate their practical performance (Figure 6a). The NVP@C cathode had a mass loading of 3.0 mg cm^−2^, resulting in a designed N/P ratio of 4.3. This corresponds to an anode utilization rate of approximately 23.3%. The full cell delivers high capacities of 99.86, 88.78, 78.78, 69.24, and 65.23 mAh g^−1^ with current densities ranging from 100 to 2000 mA g^−1^ (Figure 6b,c). Conversely, the Na@HCF||NVP@C full cell exhibits significantly lower capacities under the same conditions (Figure S23). The Na@Sb@HCF||NVP@C cell maintains a high capacity of 84.10 mA g^−1^ with a high CE of 98.05% even after 1000 cycles under 100 mA g^−1^ (Figure 6d). By comparison, the Na@HCF||NVP@C suffers from severe capacity attenuation and poor CE (Figure S24). The charge transfer kinetics were further investigated by in situ EIS measurement of the full cells. The Equivalent circuit model of the In situ EIS is showed in Figure S25. The Na@Sb@HCF ||NVP@C cell demonstrates excellent cycling stability and lower R_ct_ (Figure 6e and Table S4), whereas the Na@HCF|NVP@C cell shows irreversible cyclicality and high R_ct_ (Figure S26). In the Galvanostatic Intermittent Titration Technique (GITT) analysis, the Sb@HCF composite exhibits both a prolonged voltage plateau (Figure S27a,b) and a higher calculated Na^+^ diffusion coefficient than pristine HCF (Figure S27c), indicating faster ion transport kinetics. To demonstrate practical applications, flexible pouch cells on the Na@Sb@HCF||NVP@C were assembled. It displays a long‐term cyclability over 200 cycles with an average CE of 98.51%, and could drive an electronic clock even under mechanical folding up to 180° (Figure 6f).

## 3 Conclusion

In summary, we successfully fabricated a 3D Sb@HCF host that synergistically combines hollow carbon confinement with Sb‐derived sodiophilic alloy highways to address the key challenges of SMA. The unique architecture enables spatially controlled Na deposition, accelerated Na^+^ diffusion, and stable SEI formation, as justified by in situ TEM, XRD, electrochemical analyses, and theoretical simulations. The Na||Sb@HCF half‐cell achieves a CE of 99.88% over 1400 cycles at 4 mA cm^−2^/4 mAh cm^−2^, while symmetric cells maintain stable operation for 1200 h at 10 mA cm^−2^/2 mAh cm^−2^ with a superior cumulative capacity of 6 Ah cm^−2^. Full cells paired with NVP@C cathodes retain a capacity of 84.10 mAh g^−1^ after 1000 cycles, and flexible pouch cells also demonstrate a stable 200 cycles cycling‐life under various folded state. This work not only highlights the potential of Sb@HCF as a high‐performance anode but also provides insights into the rational design of alloy‐integrated hosts for next‐generation energy storage systems.

## Conflicts of Interest

The authors declare no conflict of interest.

## Supporting information




**Supporting File 1**: advs75088‐sup‐0001‐SuppMat.docx.

**Supporting File 2**: advs75088‐sup‐0002‐VideoS2.mp4.

**Supporting File 3**: advs75088‐sup‐0003‐VideoS2.mp4.

## Data Availability

The data that support the findings of this study are available from the corresponding author upon reasonable request.
