# Supplementary material for: Synergistic Ion Transport and Spatial Confinement in Sb‐Embedded Hollow Carbon Nanofibers for Stable Na Metal Anodes
Source: Adv Sci (Weinh). 2026 Apr 7;13(34):e21115. doi: 10.1002/advs.202521115 (PMC13285129; doi:10.1002/advs.202521115)
Supplement: Supplementary file 1 — Supporting File 1: advs75088‐sup‐0001‐SuppMat.docx. [file ADVS-13-e21115-s001.docx]

Supporting Information

Synergistic Ion Transport and Spatial Confinement in Sb-Embedded Hollow Carbon Nanofibers for Stable Na Metal Anodes

*Feng Han*^‡^*, Menghuan Yuan*^‡^*, Hui Wang^*^, Dezhi Kong, Baitao Liu, Yongtao Tian, Xinjian Li, Ye Wang, Tingting Xu^*^and Hui Ying Yang^*^*

[a] Feng Han, Menghuan Yuan, Hui Wang, De Zhi Kong, Yongtao Tian, Xinjian Li, Ye Wang, and Tingting Xu

Key Laboratory of Material Physics, Ministry of Education, School of Physic, Zhengzhou University, Zhengzhou 450001, China

Email address: [aphwang@zzu.edu.cn](mailto:aphwang@zzu.edu.cn); xutt@zzu.edu.cn

[b] Hui Ying Yang

College of Design and Engineering, National University of Singapore, 9 Engineering Drive 1, Singapore 117575.

Email address: yanghuiying@nus.edu.sg

Experimental Section/Methods

*Synthesis of HCF and Sb@HCF.* A homogeneous spinning solution was prepared by dissolving 1.8 g of polyacrylonitrile (PAN), 1.4 g of pre-synthesized silica nanospheres, and 1.8 g of SbCl_3_ in 15 mL of N,N-Dimethylformamide, followed by stirring at 75 °C for 5 hours. The resulting viscous solution was electrospun at 14 kV with a feed rate of 2 mL/h. The as-spun nanofibers were stabilized in air at 200 °C for 12 h and then carbonized at 700 °C for 10 h under a N_2_/H_2_ (10:1 v/v) atmosphere. After carbonization, the silica nanospheres were removed by etching with hydrofluoric acid, creating hollow cavities in Sb@HCF. The product was washed thoroughly with deionized water and dried. For comparison, HCF were synthesized under identical conditions without the addition of SbCl_3_.

*Materials Characterization.* The morphology of Sb@HCF and HCF fibers was characterized using field-emission scanning electron microscopy (FE-SEM, JSM-6700F, JEOL). More detailed morphological and structural analysis was conducted via transmission electron microscopy (TEM; JEM-2100, JEOL). X-ray photoelectron spectroscopy (XPS) was employed to determine key changes in the elemental composition and chemical states of the samples before and after electrochemical cycling. Cryo-TEM were performed using Thermo Scientific Titan Krios G3i (USA) operated at 300 kV, which is equipped with automatic injection system of frozen sample. The crystal structure of the Sb@HCF fibers was analyzed by X-ray powder diffraction (XRD) using Cu Kα radiation. For the ***in-situ* XRD measurements**, an assembled CR2032 coin cell was electrochemically cycled using a battery tester at a current density of 102 mA g^-1^ between 0.01 and 2 V (vs Na^+^/Na). XRD patterns **were** collected during cycling **at a scan rate** of 2.5 °/min.

*Battery fabrication.* To evaluate the electrochemical performance of Sb@HCF, HCF, and Cu foil anodes, CR2032 coin cells were assembled in an argon-filled glove box. Half-cells employed the anode material as the working electrode, coupled with a Na metal counter electrode, a Celgard 2400 separator, and an electrolyte of 1 M NaPF_6_ in diglyme. Symmetric cells were constructed with Na-preplated Na@Sb@HCF or Na@HCF electrodes for cycling stability tests. Full cells incorporated NVP@C cathodes, prepared by coating a slurry of NVP@C, carbon black, and PVDF (8:1:1 by weight) on aluminum foil, with a mass loading of 3.2 mg cm^-2^ and an N/P ratio of 5.1. All cells used a consistent electrolyte volume of 70 µL. For *in-situ* optical microscopy observation, a visualization cell equipped with a sapphire viewport was assembled within an argon-filled glove box.

*Configuration of in-situ TEM.* In-situ TEM characterization of Na plating/stripping dynamics was conducted using a JEM-2100 microscope equipped with a PicoFemto electrochemical holder. A nanobattery device was configured inside the holder by attaching an individual Sb@HCF fiber to a copper wire as the working electrode and scratched Na metal (with its native Na_2_O layer) on a tungsten tip as the counter electrode. Na plating and stripping processed were triggered by applying bias voltages to the tungsten tip.

*Theoretical simulations.* In the COMSOL simulation, Cu, HCF, and Sb@HCF fibers serve as the negative electrode with a fixed voltage under isothermal conditions (298 K). All AIMD computations employed the projector augmented-wave (PAW) method and the PBE functional, using a Γ-point sampling and a plane-wave energy cutoff of 350 eV.


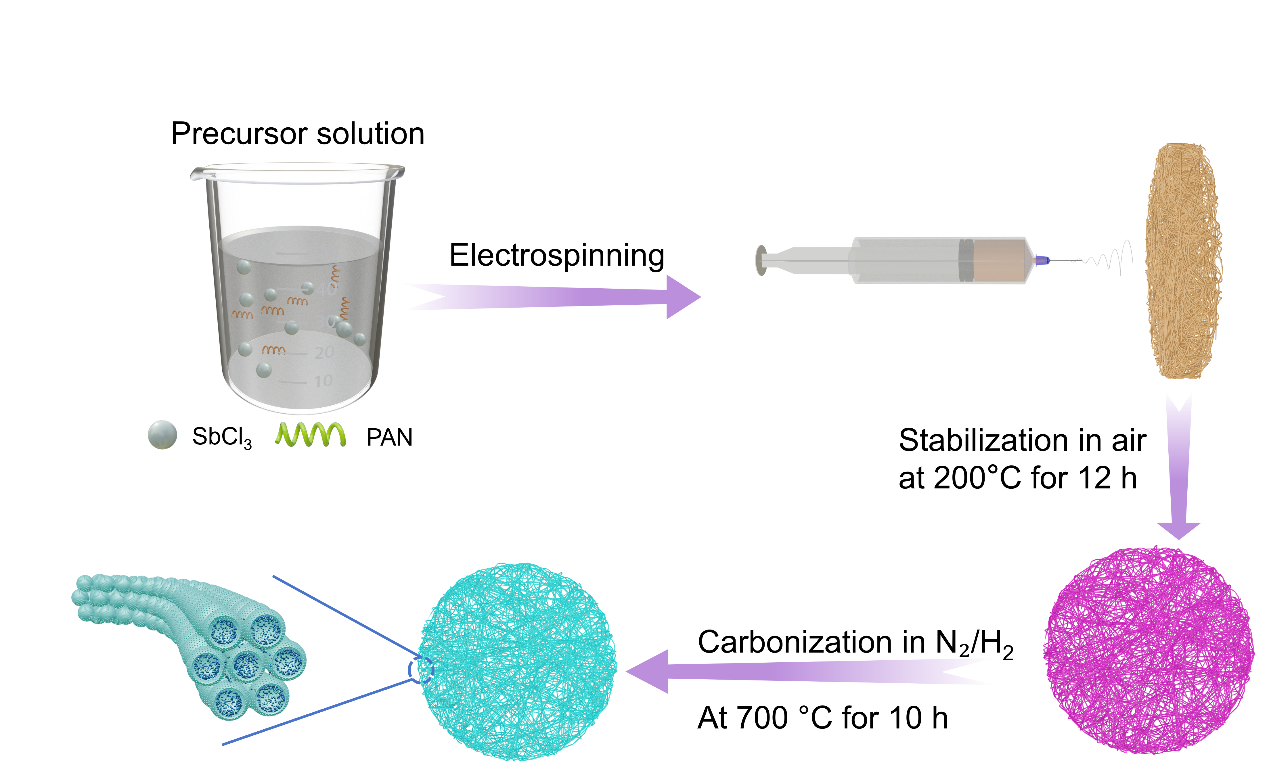


**Figure S1.** Schematic diagram of preparation process for Sb@HCF.


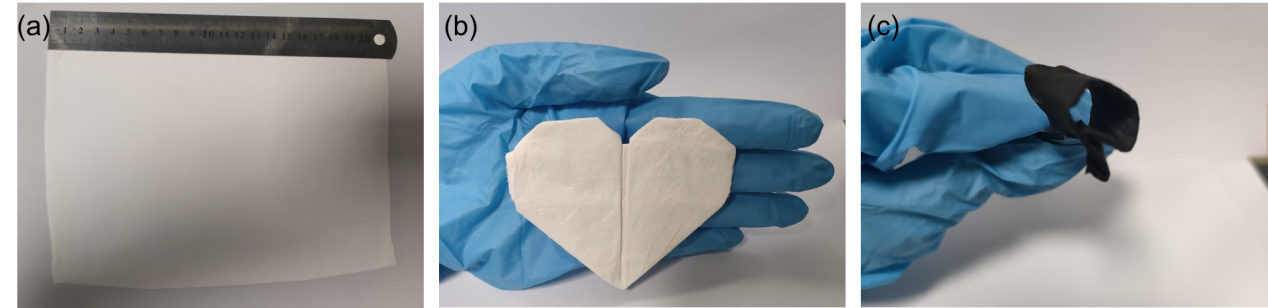


**Figure S2.** (a-b) Digital photos of Sb@HCF precursor paper. (c) The Sb@HCF precursor paper after carbonization.


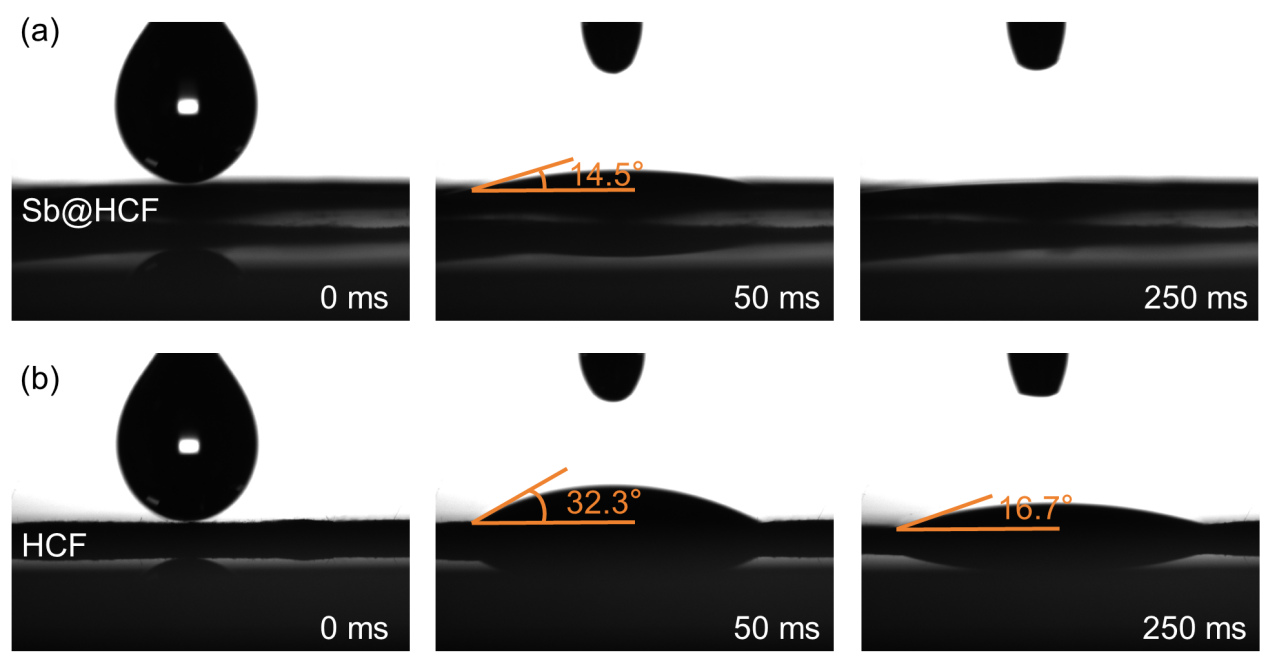


**Figure S3.** (a-b) The electrolyte wettability of Sb@HCF and HCF electrodes.


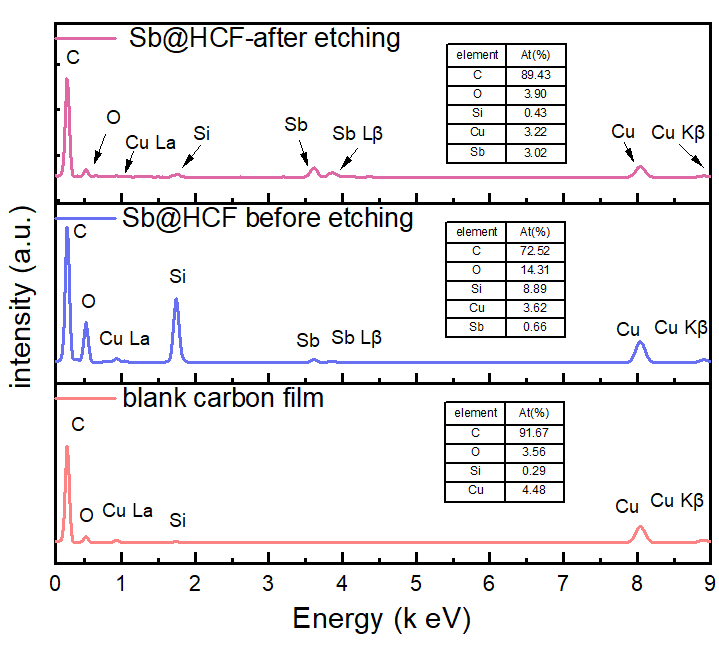


**Figure S4.** EDS data for Sb@HCF before and after HF etching, as well as for a blank carbon film on a TEM grid as a reference.


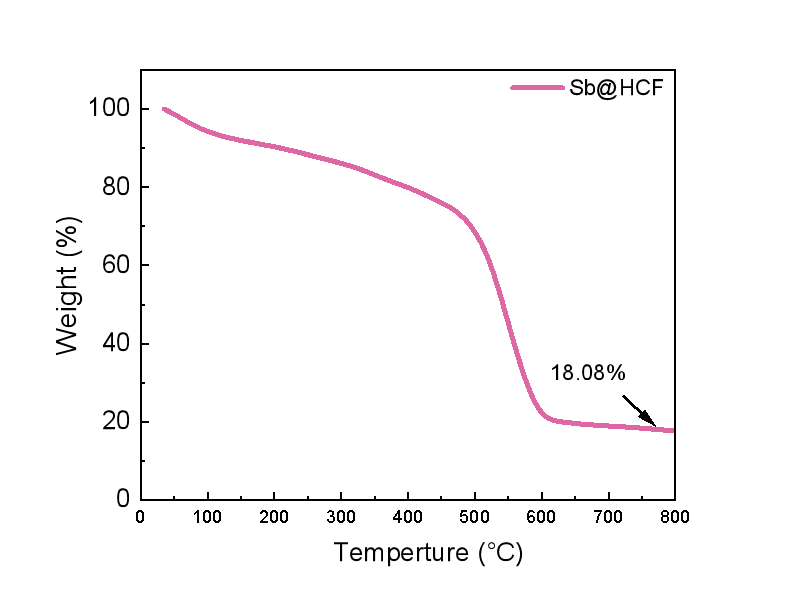


**Figure S5.** The TGA characterization of the Sb@HCF electrode.


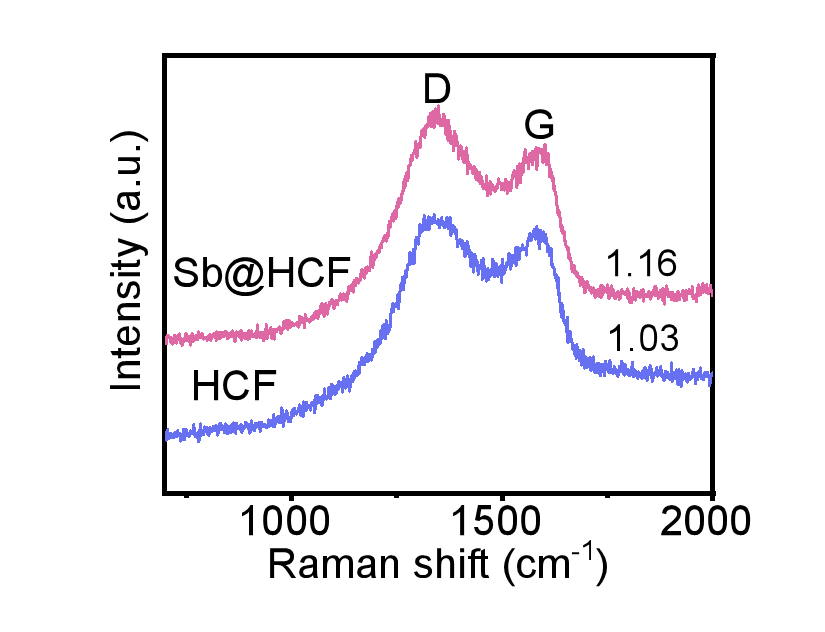


**Figure S6.** Raman analysis of Sb@HCF and HCF fiber.

**
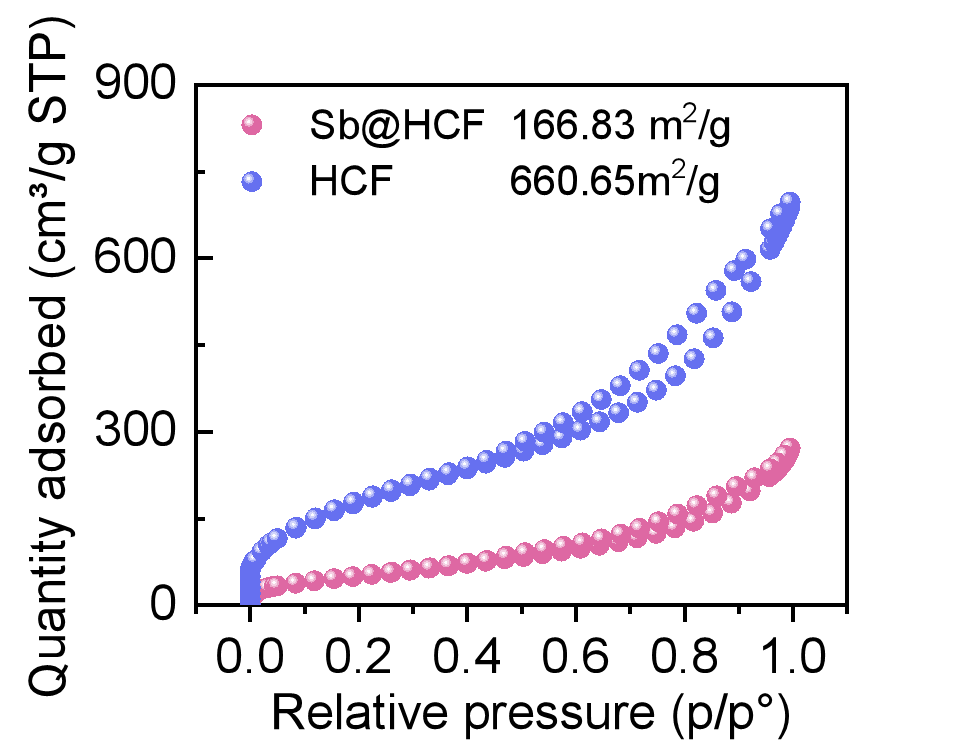
**

**Figure S7.** N_2_ adsorption–desorption isotherm of HCF and Sb@HCF.

**
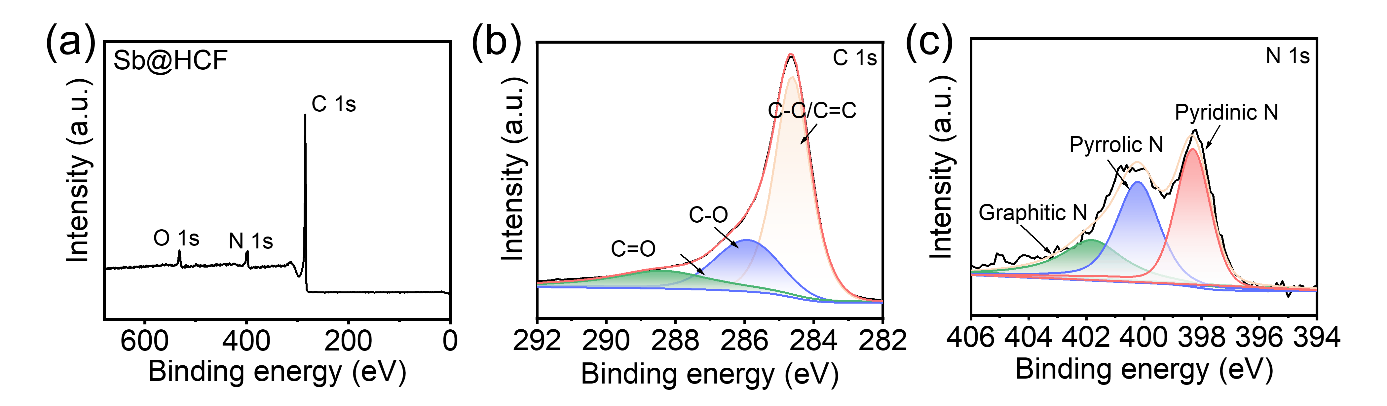
**

**Figure S8.** (a) XPS survey spectrum of Sb@HCF electrodes, (b) C 1s and (c)N 1s spectra of the pristine Sb@HCF electrodes.

**
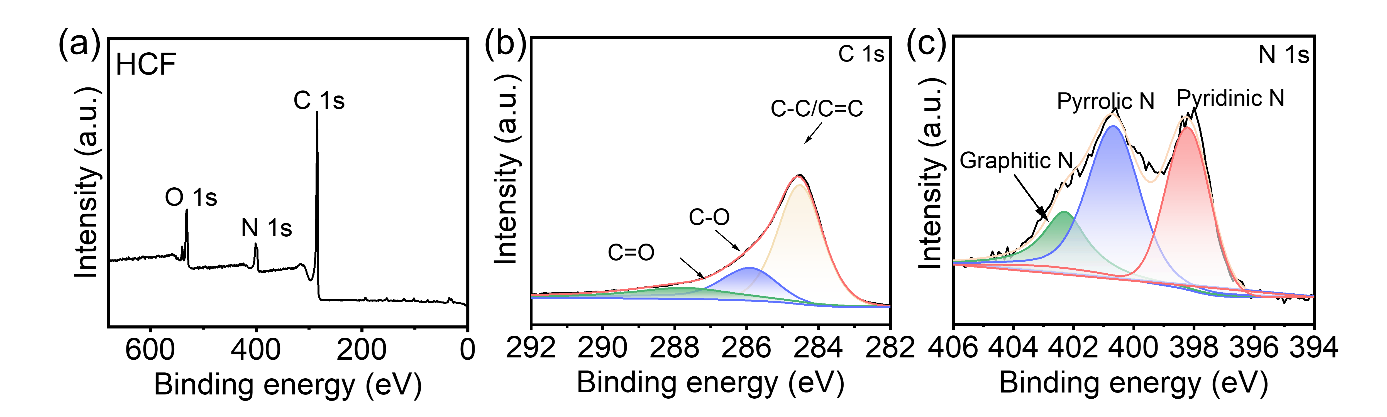
**

**Figure S9.** (a) XPS survey spectrum of HCF electrodes, (b) O 1s, (c)C 1s and (d) N 1s spectra of the pristine HCF electrodes.

**Figure S10.** Voltage profiles of the Cu electrodes at various current densities.


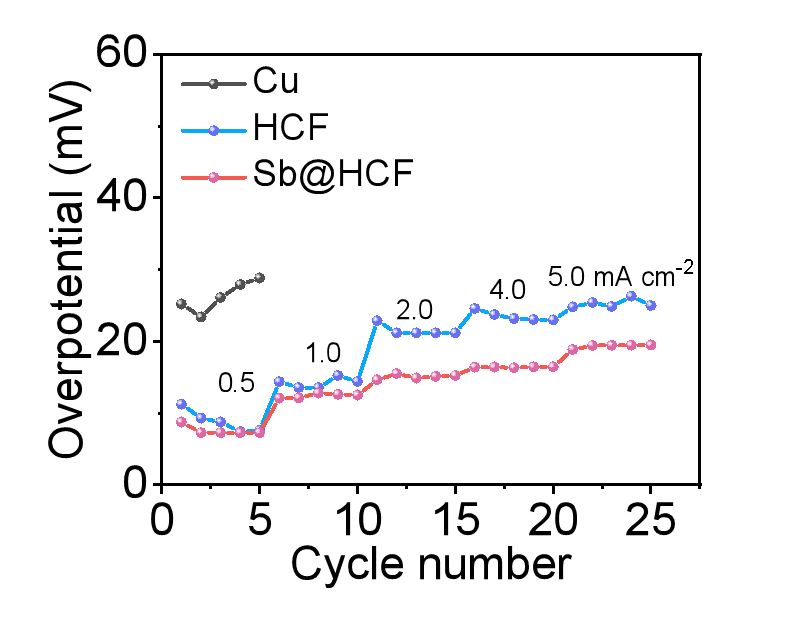


**Figure S11.** Nucleation overpotential of the Sb@HCF, HCF and Cu at different current densities.


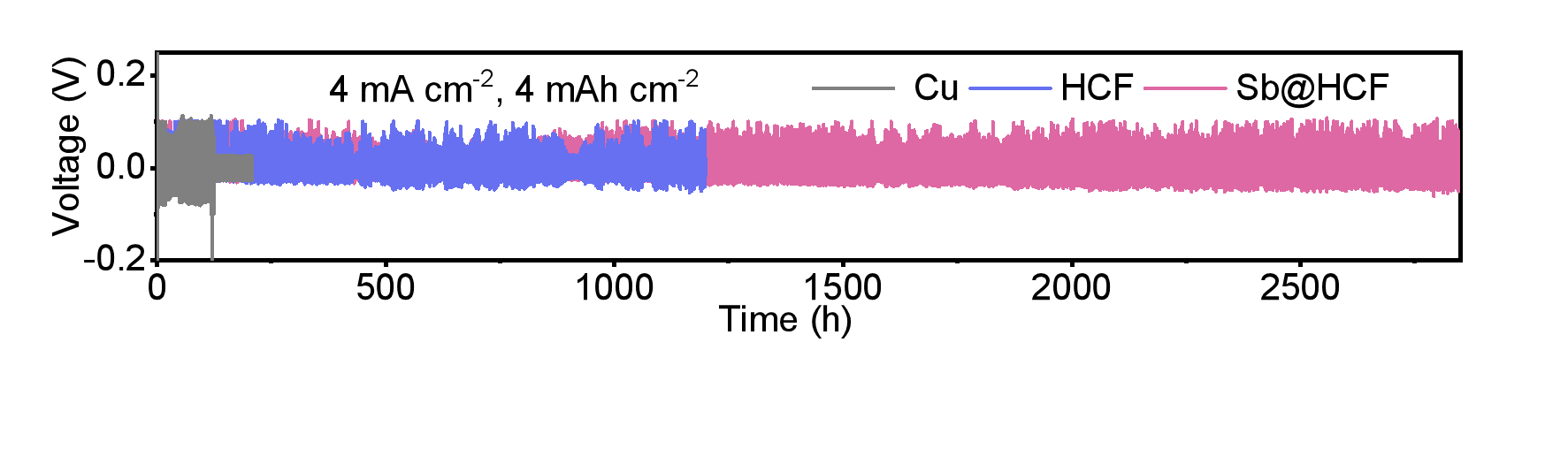


**Figure S12.** The Long-cycling performance of Sb@HCF, HCF and Cu electrodes with 4 mA cm^-2^ and a specific capacity of 4 mAh cm^-2^.


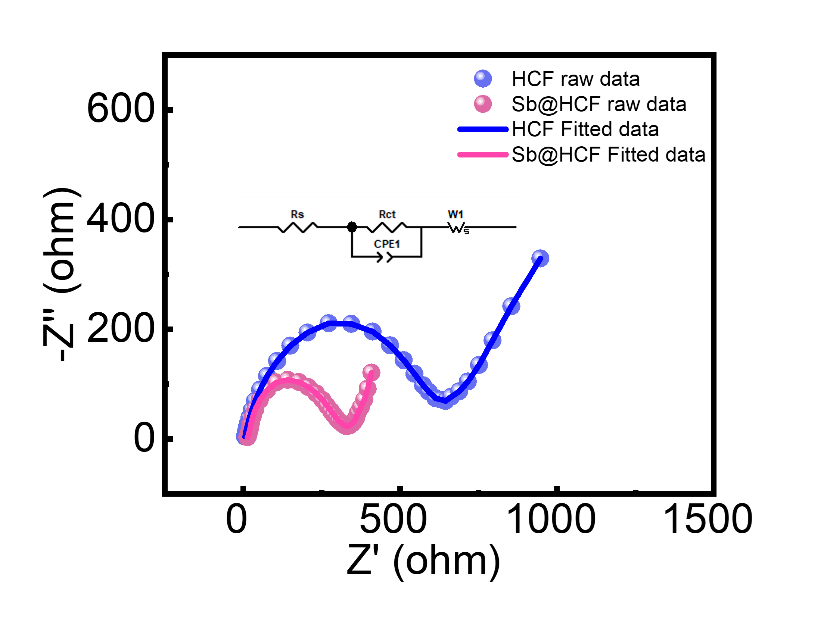


**Figure S13.** EIS curves of the Sb@HCF and HCF electrodes before cycling.


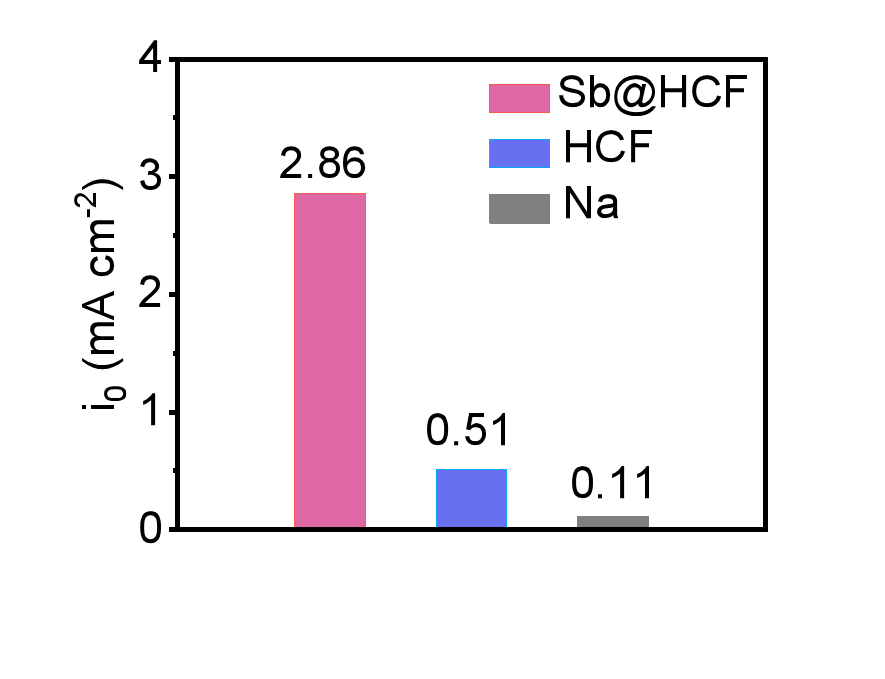


**Figure S14.** The exchange current densities of Sb@HCF, HCF and Na electrodes.


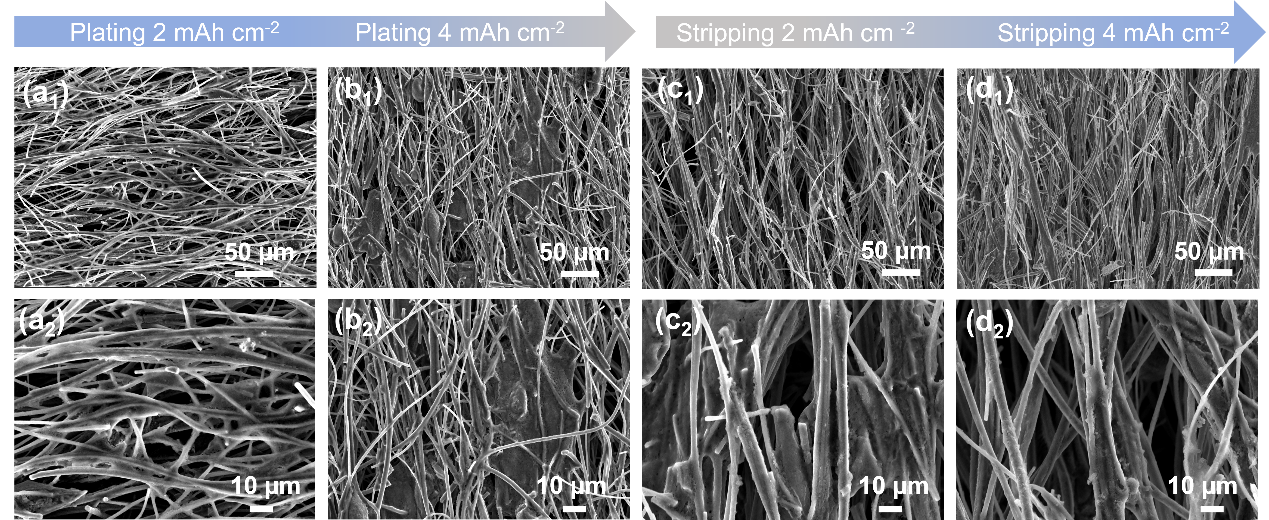


**Figure S15.** The morphology evolution of HCF and the corresponding SEM images at a plating capacity of (a_1_, a_2_) 2 mAh cm^-2^, (b_1_, b_2_) 4 mAh cm^-2^, and a stripping capacity of (c_1_, c_2_) 2 mAh cm^-2^, (e_1_, e_2_) 4 mAh cm^-2^.


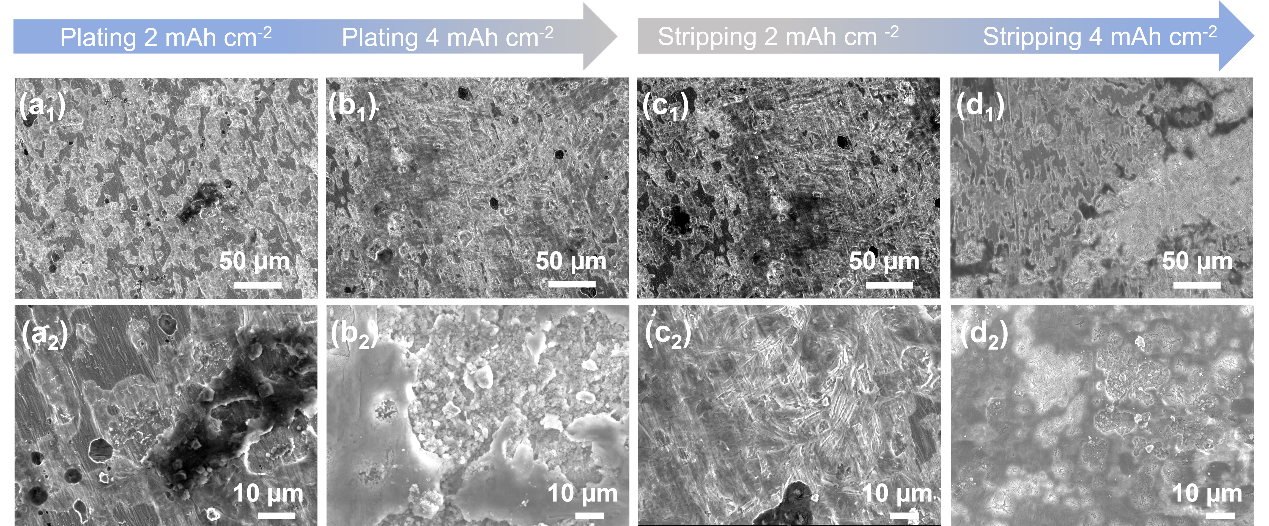


**Figure S16.** The morphology evolution of Cu and the corresponding SEM images at a plating capacity of (a_1_, a_2_) 2 mAh cm^-2^, (b_1_, b_2_) 4 mAh cm^-2^, and a stripping capacity of (c_1_, c_2_) 2 mAh cm^-2^, (e_1_, e_2_) 4 mAh cm^-2^.


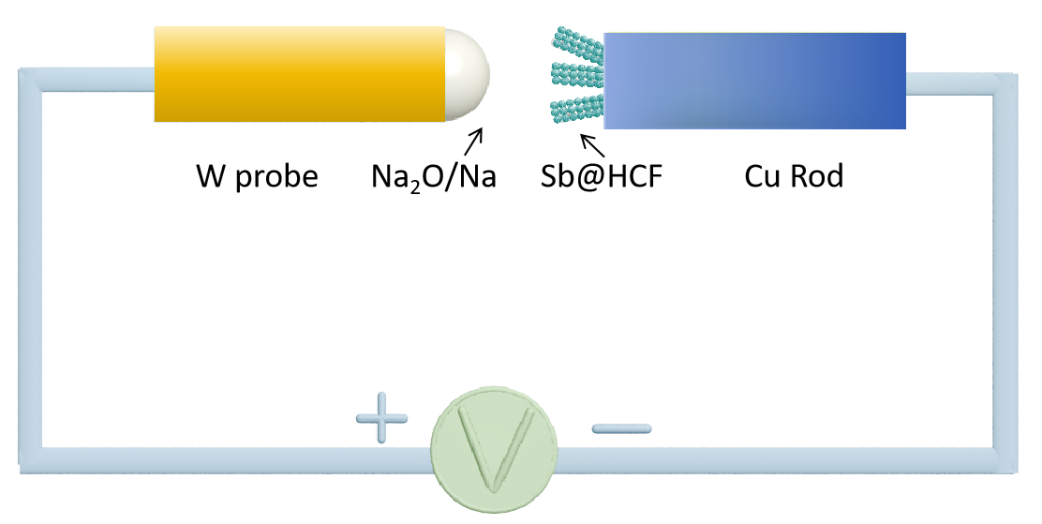


**Figure S17.** Schematic diagram of the *in-situ* constructed dry cell for Na plating in TEM.


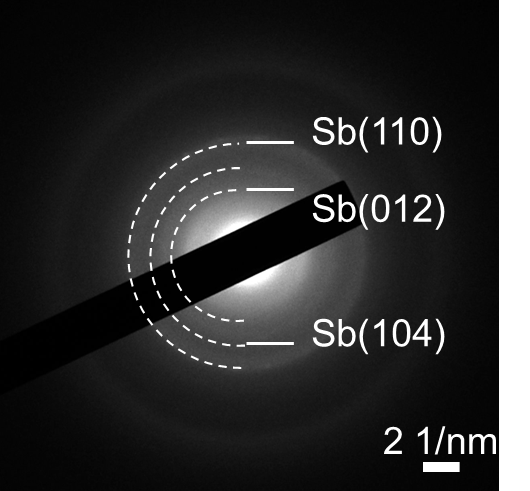


**Figure S18.**The SEAD image of the Sb@HCF.


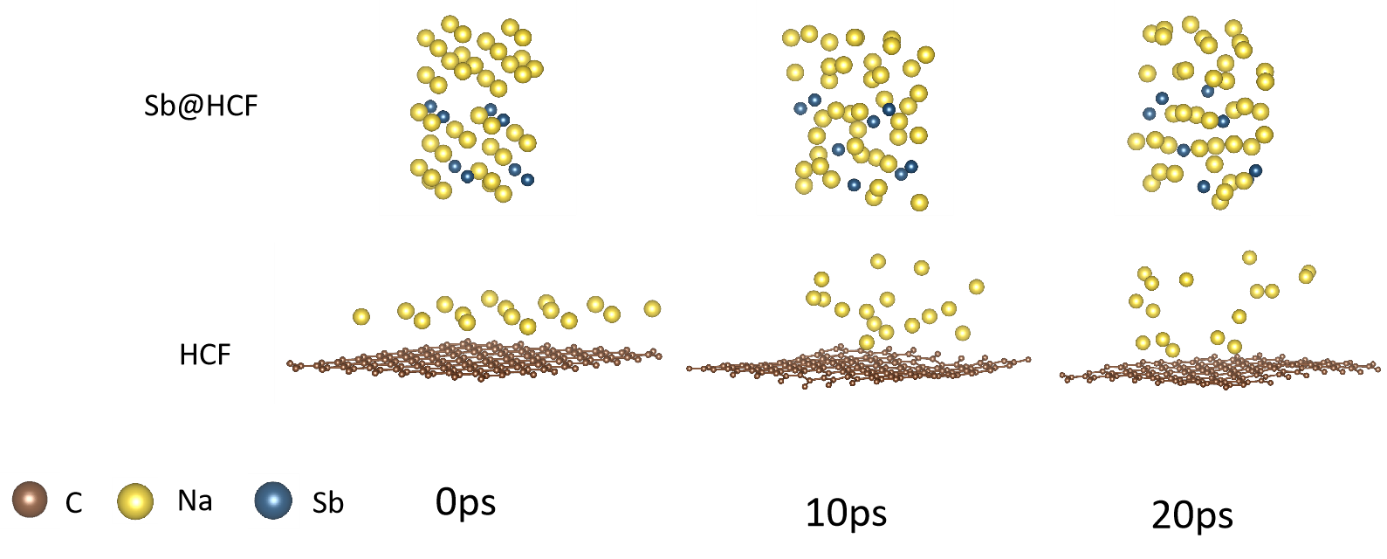


**Figure S19.** AIMD simulation snapshots of Na^+^ on Na_3_Sb alloy and carbon matrix.


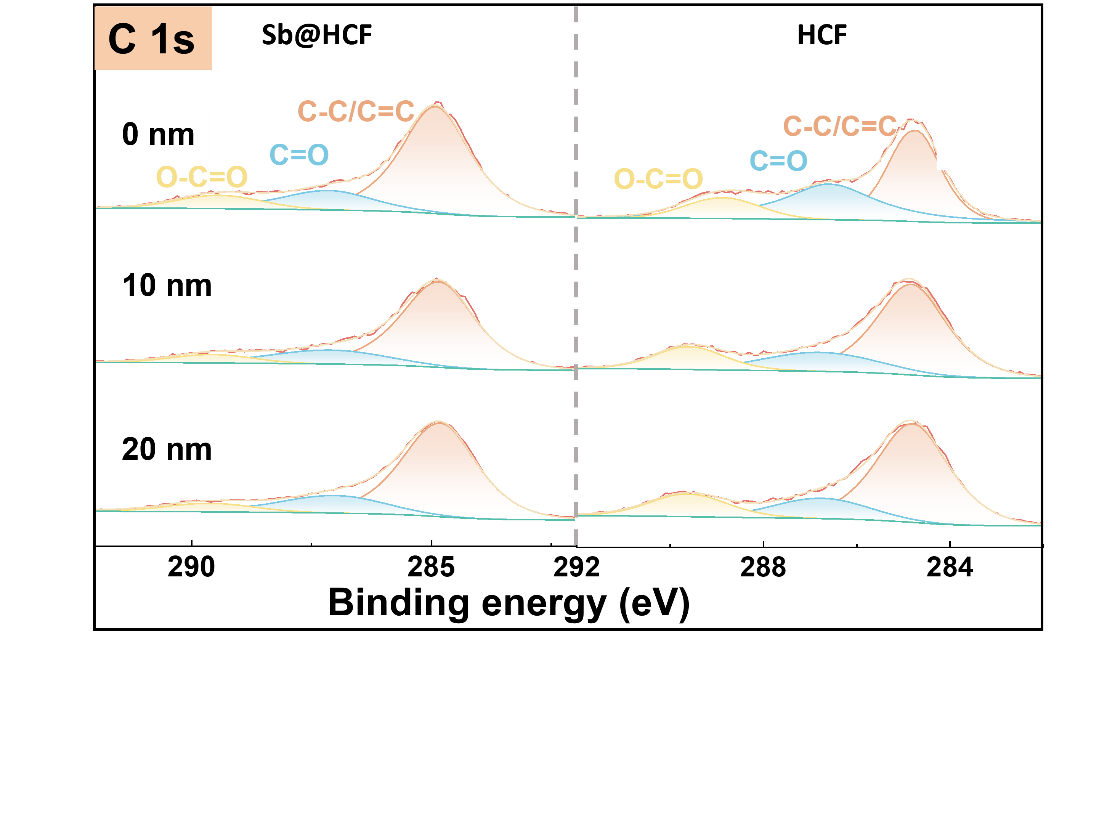


**Figure S20.** In-depth XPS spectra of (a) C 1s for Sb@HCF and HCF electrodes.


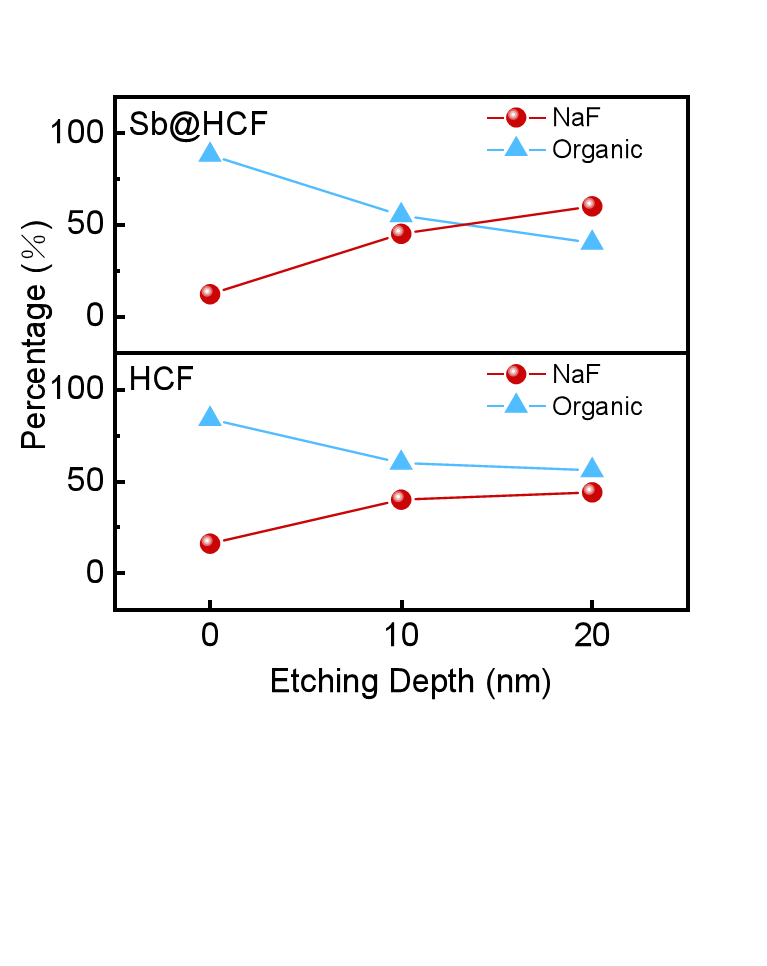


**Figure S21.** Percentage of each content for SEI in Sb@HCF anode and HCF anode.


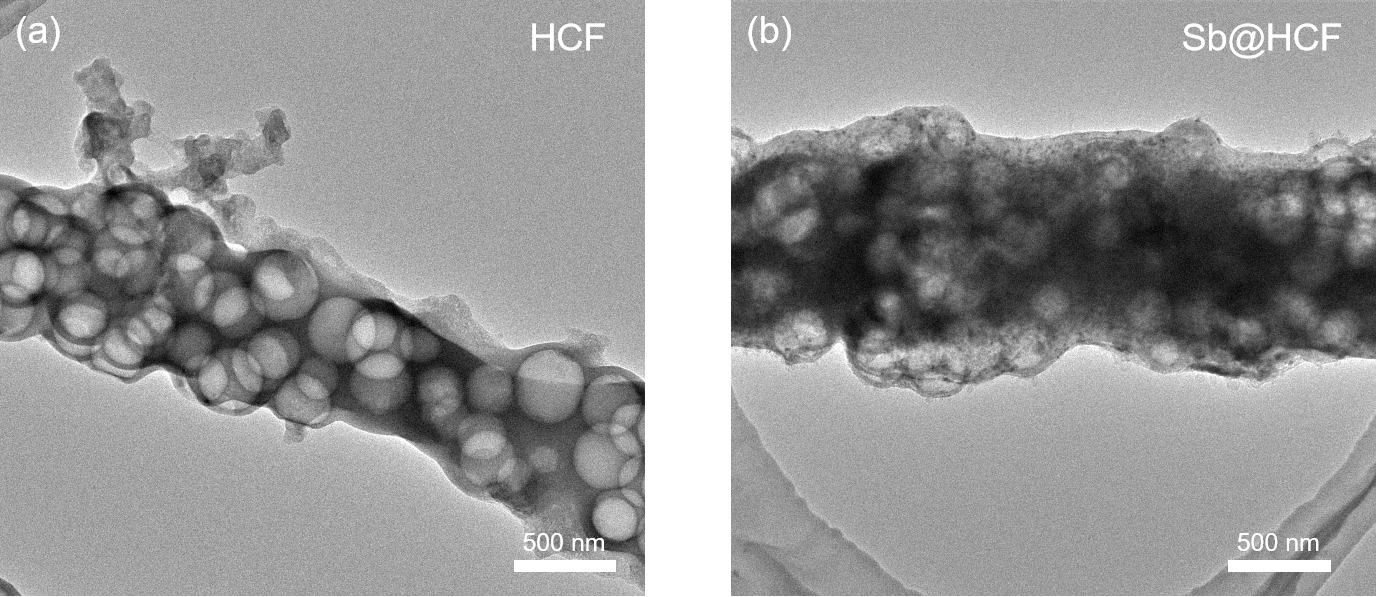


**Figure S22.** TEM images of theSb@HCF and HCF electrode surface after 75 cycles


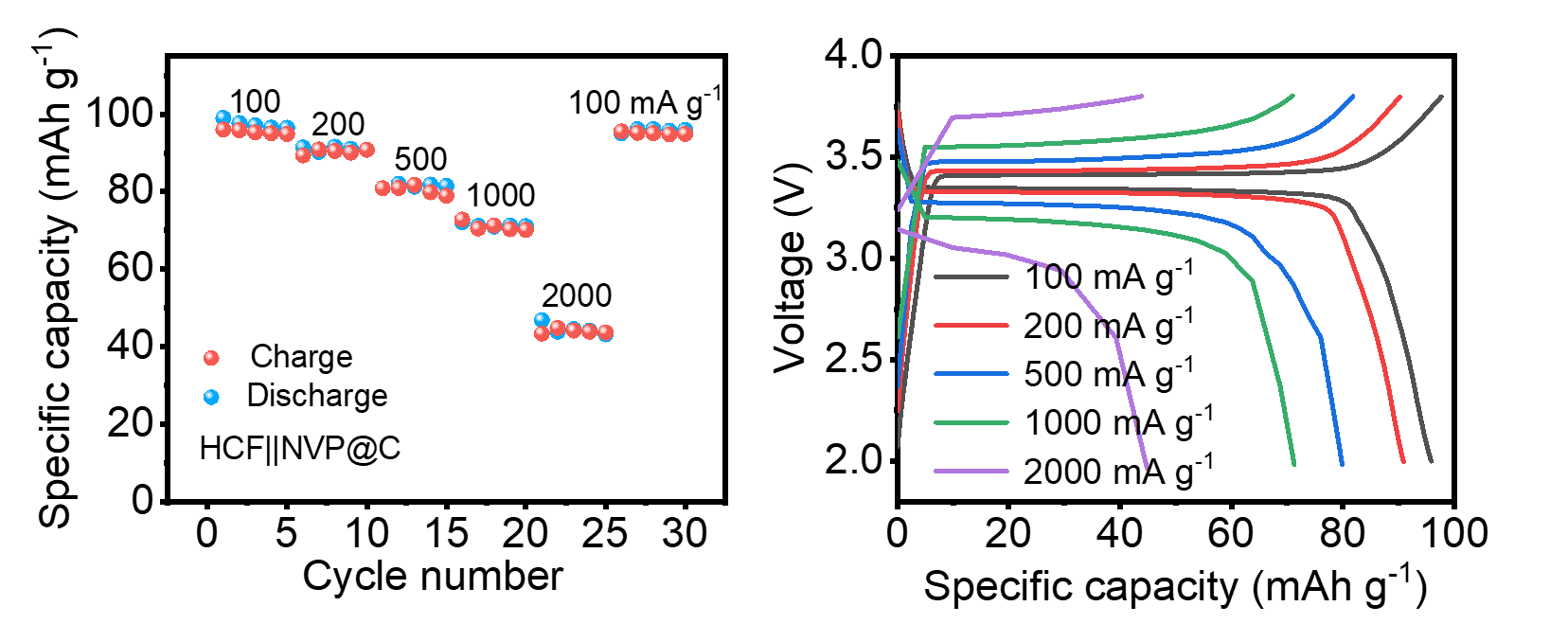


**Figure S23.** (a) Rate performance and the (b) related GCD curves at the current densities range of 100-2000 mA g^-1^ for HCF electrode.

**Figure S24.** The Cycling performance and related CE of HCF electrode at 100 mA g^-1^.


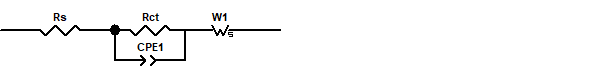


**Figure S25.** Equivalent circuit model of the *In-situ* EIS.


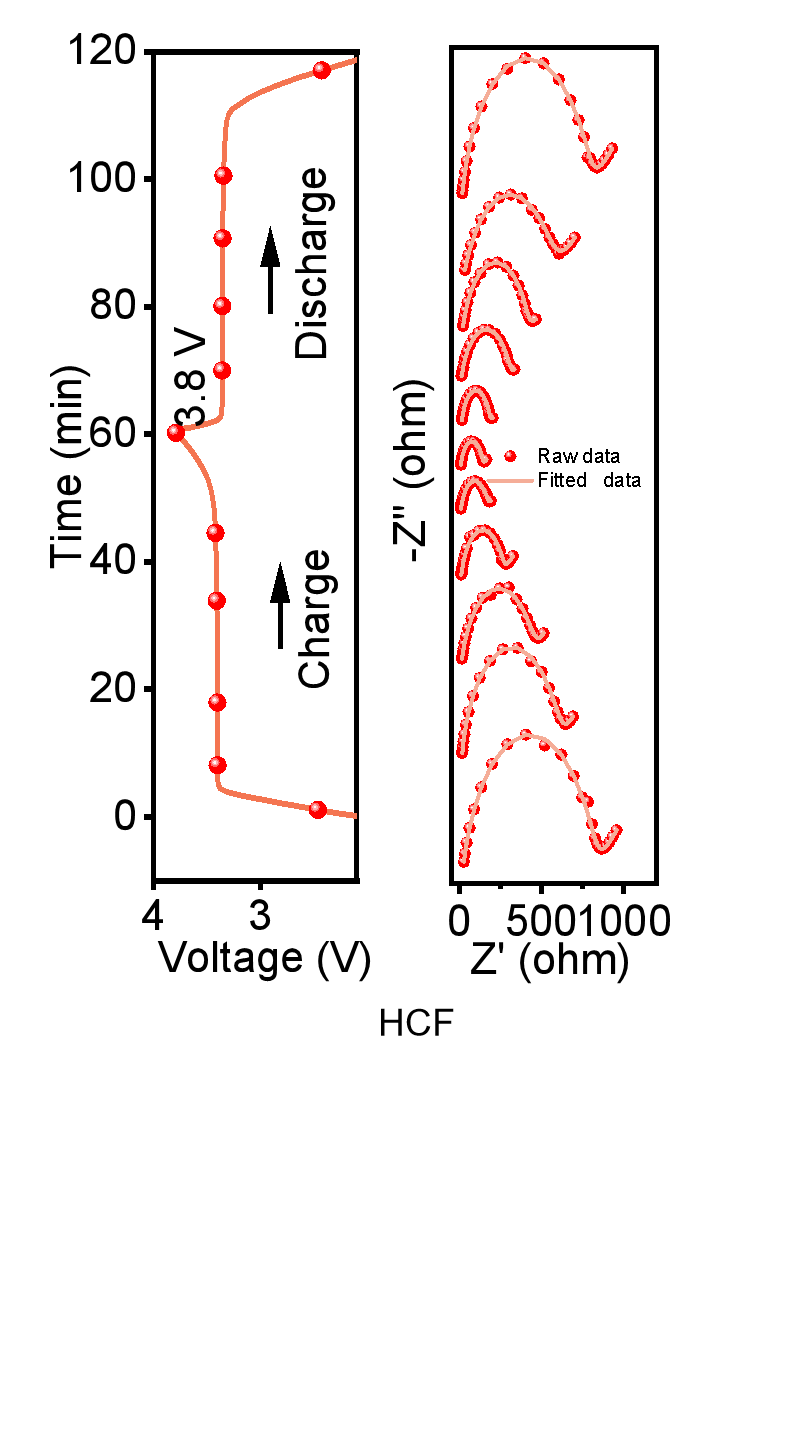


**Figure S26.** *In-situ* EIS results of the Na@HCF||NVP@C full cells.


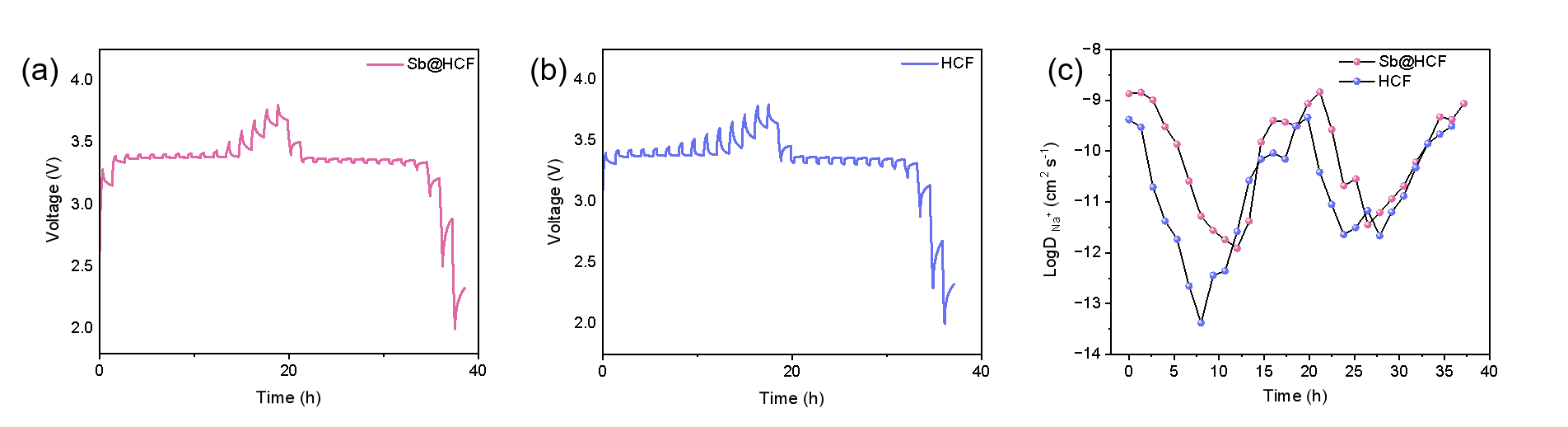


**Figure S27.** (a) GITT curves of Na@Sb@HCF||NVP@C and Na@HCF||NVP@C respectively. (b) Calculated Na ion diffusion coeffcient based on above GITT curves.

**Table S1.** EIS fitting results for Sb@HCF and HCF electrodes before cycling.

| **Electrode** | **R_S_ (Ω)**  **Before cycling** | **R_ct_ (Ω)**  **before cycling** |
| --- | --- | --- |
| Sb@HCF | 13.2 | 319.8 |
| HCF | 5.5 | 639.5 |

**Table S2.** EIS fitting results for Sb@HCF and HCF electrodes after cycling.

| **Electrode** | **R_SEI_ (Ω)**  **After 5 cycles** | **R_SEI_ (Ω)**  **After 15 cycles** | **R_ct_ (Ω)**  **After 5 cycles** | **R_ct_ (Ω)**  **After 15 cycles** |
| --- | --- | --- | --- | --- |
| Sb@HCF | 7.6 | 7.5 | 3.2 | 1.9 |
| HCF | 22.5 | 13.8 | 4.1 | 2.4 |

**Table S3.** The electrochemical performance of core-shell host structures incorporating sodiophilic sites.

| **Electrode** | **Current density**  **(mA cm^-2^)** | **Capacity (mAh cm^-2^)** | **Cycle number** | **Cumulative capacity**  **(Ah cm^-2^ × cycle)** | **Ref.** |
| --- | --- | --- | --- | --- | --- |
| **Sb@HCF** | 10 | 2 | 3000 | **6** | **This work** |
| Sb@HPCNF | 10 | 5 | 200 | 1 | ^[1]^ |
| Bi@NC | 3 | 1 | 4350 | 4.35 | ^[2]^ |
| NO-CNCF | 2 | 2 | 1200 | 2.4 | ^[3]^ |
| CMFS | 5 | 5 | 500 | 2.5 | ^[4]^ |
| CFN-CP | 5 | 1 | 750 | 0.75 | ^[5]^ |
| LCNF | 1 | 1 | 100 | 0.1 | ^[6]^ |
| SnNCNF | 10 | 10 | 350 | 3.5 | ^[7]^ |

**Table S4.** *In-situ* EIS fitting results for Na@Sb@HCF ||NVP@C and Na@HCF||NVP@C full cells.

| **State** | **Na@Sb@HCF\|\|NVP@C** | | **Na@HCF\|\|NVP@C** | |
| --- | --- | --- | --- | --- |
|  | **R_s_ (Ω)** | **R_ct_ (Ω)** | **R**_s_ (**Ω**) | **R**_ct_ (**Ω**) |
| Charge1 | 12.7 | 381.0 | 28.1 | 858.0 |
| Charge2 | 13.3 | 305.0 | 17.7 | 625.4 |
| Charge3 | 10.9 | 238.5 | 16.2 | 462.7 |
| Charge4 | 16.6 | 187.0 | 12.7 | 273.0 |
| Charge5 | 11.0 | 152.9 | 10.6 | 170.4 |
| Charge6 | 5.5 | 106.4 | 10.4 | 137.8 |
| Discharge1 | 10.8 | 143.6 | 15.5 | 168.1 |
| Discharge2 | 13.1 | 162.0 | 13.7 | 300.7 |
| Discharge3 | 11.3 | 217.9 | 22.6 | 400.9 |
| Discharge4 | 13.3 | 322.2 | 34.6 | 571.2 |
| Discharge5 | 12.5 | 399.6 | 18.6 | 805.8 |

**References:**

[1] Z. Li, H. Qin, W. Tian, L. Miao, K. Cao, Y. Si, H. Li, Q. Wang, L. Jiao, *Adv. Funct. Mater.* **2023**, *34*, 2301554.

[2] M. Yuan, H. Wang, T. Xu, N. Chu, D. Kong, L. Zeng, Y. Wang, X. Bai, H. Seok Park, *Angew. Chem., Int. Ed.* **2025**, *64*, e202417930.

[3] C. Chu, C. Wang, W. Meng, F. Cai, B. Wang, N. Wang, J. Yang, Z. Bai, *Carbon Energy.* **2024**, *6*, e601.

[4] L. Yue, Y. Qi, Y. Niu, S. Bao, M. Xu, *Adv. Energy Mater.* **2021**, *11*, 2102497.

[5] J. Xiao, N. Xiao, K. Li, L. Zhang, X. Ma, Y. Li, C. Leng, J. Qiu, *Adv. Funct. Mater.* **2022**, *32*, 2111133.

[6] L. Tao, A. Hu, L. Mu, D. J. Kautz, Z. Xu, Y. Feng, H. Huang, F. Lin, *Adv. Funct. Mater.* **2020**, *31*, 2007556.

[7] S. Li, H. Zhu, Y. Liu, Q. Wu, S. Cheng, J. Xie, *Adv. Mater.* **2023**, *35*, e2301967.
